# Bayesian Model Search for Nonstationary Periodic Time Series

**DOI:** 10.1080/01621459.2019.1623043

**Published:** 2019-07-09

**Authors:** Beniamino Hadj-Amar, Bärbel Finkenstädt Rand, Mark Fiecas, Francis Lévi, Robert Huckstepp

**Affiliations:** a Department of Statistics, University of Warwick, Coventry, UK;; b Division of Biostatistics, School of Public Health, University of Minnesota, Minneapolis, MN;; c Warwick Medical School, University of Warwick, Coventry, UK;; d School of Life Sciences, University of Warwick, Coventry, UK

**Keywords:** Bayesian spectral analysis, Change-points, Reversible-jump MCMC, Sleep apnea, Ultradian sleep cycles

## Abstract

We propose a novel Bayesian methodology for analyzing nonstationary time series that exhibit oscillatory behavior. We approximate the time series using a piecewise oscillatory model with unknown periodicities, where our goal is to estimate the change-points while simultaneously identifying the potentially changing periodicities in the data. Our proposed methodology is based on a trans-dimensional Markov chain Monte Carlo algorithm that simultaneously updates the change-points and the periodicities relevant to any segment between them. We show that the proposed methodology successfully identifies time changing oscillatory behavior in two applications which are relevant to e-Health and sleep research, namely the occurrence of ultradian oscillations in human skin temperature during the time of night rest, and the detection of instances of sleep apnea in plethysmographic respiratory traces. Supplementary materials for this article are available online.

## 1 Introduction

Identifying the periodicities present in a cyclical phenomenon allows us to gain insight into the sources of variability that drive the phenomenon. For example, respiratory traces obtained from a plethysmograph used on rodents in experimental sleep apnea research exhibit many abrupt changes in their periodic components as the rat naturally changes their breathing pattern in the course of its sleep-wake activities (Han et al. 2002; Nakamura, Fukuda, and Kuwaki 2003). Similarly, human temperature, as measured by a wearable sensing device over several days at relatively high temporal resolution, may be subject to a different periodic behavior during the night when the individual transitions between ultradian sleep stages (Carskadon and Dement 2005; Komarzynski et al. 2018). While the theory and methods for analyzing the periodicities of time series data whenever the time series is stationary are relatively well-developed, the task of modelling time series that show regime shifts in periodicity, amplitude, and phase remain challenging because the timing of changes and the relevant periodicities are usually unknown.

There have been many developments for modeling stationary oscillatory time series data. Rife and Boorstyn (1976) and Stoica et al. (1989) addressed the problem of estimating the frequencies, phases, and amplitudes of sinusoidal signals under the assumption of a known number of sinusoids, where inference is based on maximum likelihood frequency estimators. These models, however, require very long time series and a large separation in the frequencies that drive the process, which will not always be the case in practice (Djuric 1996; Andrieu and Doucet 1999). Quinn (1989), Yau and Bresler (1993), and Zhang and Wong (1993) tackled the problem of model selection on the number of sinusoidal signals by employing the Akaike information criterion (Akaike 1974) and the minimum description length principle (Rissanen 1978). Djuric (1996) showed that these procedures tend to estimate a wrong number of components when the sample size is small and the signal-to-noise ratio is low.

Bayesian approaches to modeling stationary oscillatory signals were explored for the first time by Bretthorst (1988, 1990) with applications to nuclear magnetic resonance spectroscopy. Dou and Hodgson (1995, 1996) presented a Bayesian approach that uses a Gibbs sampler to identify multiple frequencies that drive the signal. Their method required the number of frequencies to be fixed in advance, and model selection was achieved by choosing the most probable model based on the estimation of the parameters for all possible models. Bayesian model selection for stationary oscillatory signals based on posterior model probabilities were also investigated by Djuric (1996). Andrieu and Doucet (1999) introduced a more efficient reversible-jump Markov chain Monte Carlo (MCMC) method (Green 1995) that jointly tackles model selection and parameter estimation for an unknown number of stationary sinusoidal signals and avoids the computationally expensive numerical optimization of Dou and Hodgson (1995, 1996) by sampling the frequencies one-at-time via Metropolis–Hastings (M-H) steps. To the best of our knowledge, currently there is no extension of this methodology to analyze nonstationary oscillatory signals.

A formal statistical modeling framework for a specific class of nonstationary time series data, called *locally stationary* time series, was developed by Dahlhaus (1997). Extending this framework to a Bayesian setting, Rosen, Stoffer, and Wood (2009) proposed an approach to model the log of the time-varying spectral density using a mixture of smoothing splines. Rosen, Wood, and Stoffer (2012) improved on this by splitting the time series into an unknown but finite number of segments of variable lengths, thereby avoiding the need to preselect partitions, and to estimate the time-varying spectral density using a fixed number of smoothing splines. For a given partition of the time series, the likelihood function is approximated via a product of local Whittle likelihoods (Whittle 1957). The methodology was developed using a Bayesian framework and is based on the assumption that, conditional on the position and number of partitions, the time series are piecewise stationary, and the underlying spectral density for each partition is smooth over frequencies. However, exploratory analyses of the time series in both of our case studies revealed spectral densities with very sharp peaks, often at several nearby frequencies, thus invalidating the assumption that the spectral density is smooth over frequencies. In addition, the frequency location of these sharp peaks changed over time.

In this article, we propose a novel Bayesian methodology for modeling oscillatory data that show regime shifts in periodicity, amplitude and phase. In contrast to previous work our approach does not require prespecifying the number of regimes or the order of the model within a regime. We assume that, conditional on the position and number of change-points, the time series can be approximated by a piecewise changing sinusoidal regression model. The timing and number of changes are unknown, along with the number and values of relevant periodicities in each segment. We develop a reversible jump MCMC technique that jointly explores the parameter space of the change-points and sub-models for all segments.

The article is organized as follows. Sections 2 and 3 present the model, the prior specifications, and the general structure of our Bayesian approach. Sections 4 and 5 provide a detailed explanation of our sampling scheme and simulation studies to demonstrate the performance of our approach. In Section 6, we illustrate the use of our methodology in two data-rich scenarios related to sleep, circadian rhythm, and e-Health research, namely the identification of the spectral properties of experimental breathing traces arising in sleep apnea research, and the analysis of human temperature data measured over several days by a wearable sensor. We conclude and discuss our current findings in Section 7.

## 2 The Model

Consider a time series realization $y_{1},\ldots ,y_{n}$ whose periodic behavior may change at *k* unknown time-points $s_{\;(k)}=(s_{1},\ldots ,s_{k})\prime$ where *k* is also unknown. Assume that in each sub-interval $I_{j}=[s_{j-1},s_{j})$ there are *m_j_* relevant frequencies $\omega_{\;j}=(\omega_{j,\;1},\ldots ,\omega_{j,\;m_{j}})\prime$, for j=1,2,…,k+1. Setting $s_{0}=1$ and $s_{k+1}=n$, we can write the following sinusoidal model (Andrieu and Doucet 1999)

(1) $$y_{t}=\sum_{j=1}^{k+1}f\;(t,\;\beta_{\;j},\;\omega_{\;j}\;)1_{\left[\;t\;\in \;I_{j}\;\right]}+\varepsilon_{t},$$

where

(2) $$\begin{array}{c} f\;(t,\;\beta_{\;j},\;\omega_{\;j}\;)=\alpha_{j}+\mu_{j}\;t\\ \;+\sum_{l=1}^{m_{j}}(\beta_{j,\;l}^{\;(1)}\;\text{cos}\;(2\pi \omega_{j,\;l}\;t)+\beta_{j,\;l}^{\;(2)}\;\text{sin}\;(2\pi \omega_{j,\;l}\;t)), \end{array}$$

$\beta_{\;j}=(\alpha_{j},\mu_{\;j},\;\beta \prime_{j,\;1},\ldots ,\;\beta \prime_{j,\;m_{j}})\prime$, $\beta_{j,\;l}=(\;\beta_{j,l}^{\;(1)},\;\beta_{j,l}^{\;(2)}\;)\prime$, $1_{\left[\cdot \right]}$ denotes the indicator function, and *μ_j_* and *α_j_* may, if needed, account for a linear trend within each segment. For simplicity we assume independent zero-mean Gaussian errors with regime-specific variances

(3) $$\varepsilon_{t}∼N\;(0,\sigma_{j}^{2}),\;\text{for}\;\;t\in I_{j}\;\;\;\text{and}\;\;\;j=1,\ldots ,k+1,$$

noting that the methodology can in principle be extended to the non-Gaussian case.

The dimension of the model is given by the number of change-points *k* and the number of frequency components in each regime denoted by $m_{\;(k)}=(m_{1},\ldots ,m_{k+1})\prime$. Furthermore, let $\beta_{\;(k)}=(\;\beta \prime_{\;1},\ldots ,\beta \prime_{\;k+1})\prime ,\omega_{\;(k)}=(\omega \prime_{\;1},\ldots ,\omega \prime_{\;k+1})\prime$, $\sigma_{\;(k)}^{\;2}=(\sigma_{\;1}^{2},\ldots ,\sigma_{\;k+1}^{\;2})\prime$, and $\theta_{\;(k)}=(\;\beta \prime_{\;(k)},\;\omega \prime_{\;(k)},\;\sigma_{\;(k)}^{\;2\prime }\;)\prime$. Using Equation (1), the likelihood of $\left(\;k,\;m_{\;(k)},\;s_{\;(k)},\theta_{\;(k)}\;\right)$ given the data $y=(\;y_{1},\ldots ,y_{n}\;)\prime$ is

(4) $$\begin{array}{c} L(\;k,\;m_{\;(k)},\;s_{\;(k)},\;\theta_{\;(k)},\;|\;y\;)=\prod_{j=1}^{k+1}L(\;m_{j},\;\theta_{\;j}\;|\;y_{j}\;),\\ y_{j}=(\;y_{t}:\;t\;\in \;I_{j}\;), \end{array}$$

where

(5) $$\begin{array}{c} L(\;m_{j},\;\theta_{\;j}\;|\;y_{j}\;)={\left(\;2\pi \sigma_{j}^{2}\;\right)}^{\;-n_{j}/2}\\ \;\times \;\text{exp}\;\left[-\frac{1}{2\sigma_{j}^{2}}\;\sum_{\;t\;\in \;I_{j}}^{}{\left\{\;y_{t}-x_{t}\;(\omega_{j})\prime \;\beta_{\;j}\;\right\}}^{\;2}\right], \end{array}$$

$\theta_{\;j}=(\;\beta \prime_{j},\;\omega \prime_{j},\;\sigma_{\;j}^{\;2\prime }\;)\prime$ is the vector of parameters, *n_j_* the number of observations of the *j*th segment, and the vector of basis functions $x_{t}\;(\;\omega_{j}\;)$ is defined as

$$\begin{array}{c} x_{t}\;(\omega_{j})=(1,\;t,\;\;\text{cos}\;(2\pi \omega_{j,1}t),\;\;\text{sin}\;(2\pi \omega_{j,1}t),\ldots ,\\ \;\;\text{cos}\;(2\pi \omega_{j,m_{j}}t),\;\;\text{sin}\;(2\pi \omega_{j,m_{j}}t))\prime . \end{array}$$

## 3 Bayesian Inference

Given some prefixed maximal numbers of change-points, $k_{\text{max}}$, and frequencies per regime, $m_{\text{max}}$, inference is achieved by assuming that the true model is unknown but comes from a finite class of models where each model $M_{k}$, with *k* change-points, is parameterized by the vector

$$(\;m_{\;(k)},\;s_{\;(k)},\;\theta_{\;(k)}\;)\in \;\Pi_{k},\;\Pi_{k}\in \;\Pi .$$

Let $S_{k}=\{\;s_{\;(k)}\in {\left[1,\;n\right]}^{\;k}\;:\;1_{\left[1<s_{1}<\cdots <s_{k}<n\right]}\;\}$ and $\Omega_{m_{j}}={\left(0,0.5\right)}^{\;m_{j}}$ denote, respectively, the sample space for the locations of change-points and the frequencies of the *j*th segment. The overall parameter space can be written as a finite union of subspaces

$$\begin{array}{c} \Pi =\overset{k_{\text{max}}}{\underset{k=0}{\cup }}\{\;k\;\}\times \Pi_{k},\;\text{and}\\ \Pi_{k}=S_{k}\;\times \;\prod_{j=1}^{k+1}\{m_{j}\}\;\times \;\overset{m_{\text{max}}}{\underset{m_{j}=1}{\cup }}\{\;R^{2m_{j}+2}\times \Omega_{m_{j}}\times \;R^{+}\}. \end{array}$$

Bayesian inference on $k,\;m_{\;(k)},s_{\;(k)}$ and $\theta_{\;(k)}$ is based on the following factorization of the joint posterior distribution

$$\begin{array}{c} \pi \;(\;k,\;m_{\;(k)},\;s_{\;(k)},\;\theta_{\;(k)}\;|\;y\;)=\pi \;(k\;|\;y)\;\pi \;(m_{\;(k)}\;|\;k,\;y)\\ \;\pi \;(s_{\;(k)}\;|\;m_{\;(k)},\;k,\;y)\;\pi \;(\theta_{\;(k)}\;|\;s_{\;(k)},\;k,\;m_{\;(k)},\;y), \end{array}$$

where we use π ( · ) as generic notation for probability density or mass function, whichever is appropriate. Sampling from it poses a multiple model selection problem, namely of the number of change-points and number of frequencies in each regime, which can be addressed by constructing a reversible-jump MCMC algorithm Green (1995). The algorithm in its basic structure iterates between the following two moves:

1. *Segment model move:* Given a partition of the data at *k* locations $s_{\;(k)}$, inference on the parameters $m_{\;(k)}$ and $\theta_{\;(k)}$ is based on the conditional posterior
  $$\pi \;(m_{\;(k)},\;\theta_{\;(k)}\;|\;k,\;s_{\left(k\right)},\;y)=\prod_{j=1}^{k+1}\pi \;(m_{j},\;\theta_{\;j}\;|\;k,\;s_{\;(k)},\;y_{j}).$$
  A reversible-jump MCMC algorithm is performed in parallel on each of the *k* + 1 segments, where at each iteration the number of sinusoids *m_j_*, the linear coefficients $\beta_{\;j}$, the frequencies $\omega_{\;j}$, and the residual variances $\sigma_{j}^{2}$ are sampled independently in each segment, for j=1,…,k+1. Notice that at this stage the algorithm will explore subspaces of variable dimensionality regarding the number of frequencies per segment, while the change-point model remains fixed.
2. *Change-point model move:* This step performs a reversible-jump MCMC algorithm for change-point model search where the number *k* and locations of change-points $s_{\;(k)}$ are sampled, along with the linear coefficients, number of frequencies and their values as well as the residual variances for any segments affected by the move.

Our prior specifications assume independent Poisson distributions for the number of break-points *k* and frequencies in each segment *m_j_*, conditioned on $k\leq k_{\text{max}}$ and $1\leq m_{j}\leq m_{\text{max}}$, respectively. Given *k*, a prior distribution for the positions of the change-points $s_{\;(k)}$ can be chosen as in Green (1995)

(6) $$\begin{array}{c} \pi \;(s_{\;(k)}\;|\;k)=\frac{(2k+1)!}{{\left(n-1\right)}^{2k+1}}\prod_{j=0}^{k}(s_{j+1}-s_{j})\;1_{\left[s_{0}<s_{1}<\cdots <s_{k}<n\right]},\\ \;s_{0}=1,s_{k+1}=n. \end{array}$$

Conditional on *k* and $m_{\;(k)}$, we choose a uniform prior for the frequencies $\omega_{j,\;l}∼\text{Uniform}(0,0.5),\;l=1,\ldots ,m_{j},\;\;\;\text{and}\;\;\;j=1,\ldots ,k+1.$ Analogous to a Bayesian regression (Bishop 2006), a zero-mean isotropic Gaussian prior is assumed for the coefficients of the *j*th segment, $\beta_{\;j}∼N_{2m_{j}+2}(\;0,\;\sigma_{\beta }^{2}\;I\;),\;j=1,\ldots ,k+1,$ where $\sigma_{\beta }^{2}$ is a prespecified large value, and the prior on the residual variance $\sigma_{j}^{2}$ of the *j*th partition is $\text{Inverse}-\text{Gamma}\;(\frac{\nu_{0}}{2},\frac{\gamma_{0}}{2})$, where *η*_0_ and *ν*_0_ are fixed at small values.

## 4 Sampling Scheme for Nonstationary Periodic Processes

Here we provide the sampling scheme associated with the nonstationary periodic processes that we wish to model. An outline of the overall procedure is as follows. Start with an initial configuration of number of change-points *k*, along with their locations $s_{\;(k)}$; this yields a partition of the data $y=(y_{1},\ldots ,y_{k+1})$. Initialize the number of frequencies in each regime $m_{\;(k)}$ and their values $\omega_{\;(k)}$, along with the coefficients $\beta_{\;(k)}$ and residual variances $\sigma_{\;(k)}^{2}.$ At each iteration of the algorithm a segment model and a change-point model move are estimated. A random choice with probabilities (7) based on the current number of parameters will determine whether to attempt a birth, death or a within-model move. In particular, let *z* denote the current number of parameters, that is, change-points *k* in the change-point model or frequencies *m_j_* in the *j*th segment model; then, the dimension may increase by one (*birth step*) with probability *b_z_*, decrease by one (*death step*) with probability *d_z_* or remain unchanged (*within step*) with probability $\mu_{z}=1-b_{z}-d_{z}$, where

(7) $$b_{z}=c\;\text{min}\{1,\frac{\pi \;(z+1)}{\pi \;(z)}\},\;d_{z+1}=c\;\text{min}\{1,\frac{\pi \;(z)}{\pi \;(z+1)}\},$$

for some constant $c\in [0,\frac{1}{2}]$, and π (z) is the prior probability of the model including *z*. Reversibility of the Markov chain is guaranteed for move types that involve a change in dimensionality as $b_{z}\;\pi \;(z)=d_{z+1}\;\pi \;(z+1).$ Here we chose *c* = 0.4 but other values are legitimate as long as *c* is not larger than 0.5, to assure that the sum of the probabilities does not exceed 1 for some values of *z*. Naturally, $b_{k=k_{\text{max}}}=b_{m=m_{\text{max}}}=0$ and $d_{k=0}=d_{m=1}=0$. The pseudocode of the overall algorithm that describes an iteration of the sampler is given in Algorithm 1. We next describe in more detail the specific procedures needed to update the moves.

**Algorithm 1:**

1. For each segment j=1,…,k+1, perform a segment model move (Section 4.1)

Draw U∼Uniform (0,1)

**if** $U\leq b_{m_{j}}\;\to \;$ *birth-step*

**else if** $b_{m_{j}}\leq U\leq d_{m_{j}}\;\to \;$ *death-step*

**else**  →  *within-step*

2. Perform a change-point model move (Section 4.2):

Draw U∼Uniform (0,1)

**if** $U\leq b_{k}\;\to \;$ *birth-step*

**else if** $b_{k}\leq U\leq d_{k}\;\to \;$ *death-step*

**else**  →  *within-step*

### 4.1 Updating a Segment Model

Given the number of change-points *k* and their locations $s_{\;(k)}$, a segment model move is performed independently and in parallel on each of the *k* + 1 partitions. Hence, throughout this subsection the subscript relating to the *j*th segment may be dropped and a segment of interest is denoted by $y=(y_{a},\ldots ,y_{b})\prime$, which contains *n* observations. Assume that the current number of frequencies is set at *m*; then, an independent random choice is made between attempting a birth, death or within-model step, with probabilities given in (7). An outline of these moves is as follows (further details are provided in the Appendix).

#### 4.1.1 Within-Model Move

Conditioned on the number of frequencies *m*, we sample the vector of frequencies ω following Andrieu and Doucet (1999), that is, by sampling the frequencies one-at-time using a mixture of M-H steps, with target distribution

(8) $$\begin{array}{c} \pi \;(\omega \;|\;\beta ,\;\sigma^{2},\;m,\;y)\\ \;\propto \;\text{exp}\;\left[-\frac{1}{2\sigma^{2}}\sum_{t\;=\;a}^{b}\{y_{t}-x_{t}\;(\;\omega \;)\prime \;\beta \;{\}}^{2}\right]1_{[\;\omega \;\in \;\Omega_{m}]\;}. \end{array}$$

In particular, the proposal distribution is a combination of a normal random walk centered around the current frequency and a sample from values of the discrete Fourier transform of ***y***. The corresponding vector of linear parameters β is then updated in a M-H step, from the target posterior conditional distribution

(9) $$\begin{array}{c} \pi \;(\;\beta \;|\;\omega ,\;\sigma^{2},\;m,\;y)\\ \;\propto \;\text{exp}\;\left[-\frac{1}{2\sigma^{2}}\sum_{t\;=\;a}^{b}\{y_{t}-x_{t}\;(\;\omega \;)\prime \;\beta \;{\}}^{2}-\frac{1}{2\sigma_{\beta }^{2}}\;\beta \prime \beta \right], \end{array}$$

where the proposed values are drawn from normal approximations to their posterior conditional distribution. Finally, the residual variance σ^2^ is then updated in a Gibbs step from

(10) $$\begin{array}{c} \sigma_{|\;\omega ,\;\;\beta }^{2}∼\text{Inverse}-\text{Gamma}\\ \;\left(\;\frac{n+\nu_{0}}{2},\;\frac{\gamma_{0}+\sum_{t\;=\;a}^{b}\{\;y_{t}-x_{t}\;(\;\omega \;)\prime \;\beta \;{\}}^{2}}{2}\right). \end{array}$$

#### 4.1.2 Between-Model Moves

For this type of move, the number of frequencies is either proposed to increase by one (birth) or decrease by one (death). If a birth move is proposed, we have that $m^{\;p}=m^{\;c}+1$, where current and proposed values are denoted by the superscripts *c* and *p*, respectively. The proposed vector of frequencies is constructed by proposing an additional frequency to include in the current vector. Conditional on the frequencies, the corresponding vector of linear coefficients and the residual variance are sampled as in the within-model move. If a death move is proposed, we have that $m^{p}=m^{c}-1$. Hence, one of the current frequencies is randomly chosen to be removed. The proposed corresponding vector of linear coefficients is drawn, along with the residual variance. For both moves, the updates are jointly accepted or rejected in a M-H step.

### 4.2 Updating the Change-Point Model

This part of the algorithm identifies the number and locations of change-points. Suppose the number of change-points is currently set to some value *k*, then according to the probabilities given in (7) a random decision is made between adding, removing, or moving a change-point. The rules for updating these types of moves are described below and more details are given in the Appendix.

#### 4.2.1 Within-Model Move

An existing change-point is proposed to be relocated with probability $\frac{1}{k}$, obtaining say $s_{j}^{\;c}$. The update for the selected change-point is proposed from a mixture of a normal random walk centered on the current change-point $s_{j}^{\;c}$ and a sample from a uniform distribution on the interval $\left[s_{j-1}^{\;c}+\psi_{s},s_{j+1}^{\;c}-\psi_{s}\right]$. Here, we introduced *ψ_s_* as a fixed minimum time between change-points avoiding change-points being too close to each other. Rosen, Wood, and Stoffer (2012) used a similar scheme, but on a discrete-scale. The number of frequencies and their values are kept fixed, and, conditional on the relocation, the linear coefficients for the segments affected by the relocation are sampled. These updates are jointly accepted or rejected in a M-H step and the residual variances are updated in a Gibbs step.

#### 4.2.2 Between-Model Moves

For this type of move, the number of change-points may either increase (birth) or decrease (death) by one. If a birth move is proposed, we have that $k^{\;p}=k^{\;c}+1$. The new proposed change-point is drawn uniformly on $f\;(s_{\;(k^{\;\;c})}^{\;c},\;\psi_{s})$, the support of $s_{\;(k^{\;\;c})}^{\;c}$ given the constraints imposed by *ψ_s_*, that is, $f\;(s_{\;(k^{\;\;c})}^{\;c},\;\psi_{s})=[1+\psi_{s},\;s_{1}^{\;c}-\psi_{s}]\;\cup \;[s_{1}^{\;c}+\psi_{s},\;s_{2}^{\;c}-\psi_{s}]\cup \;\cdots \;\cup \;[s_{k^{\;\;c}}^{\;c}+\psi_{s},\;n-\psi_{s}]$. The latter involves splitting an existing segment. The number of frequencies and their values in the proposed segments are selected from the current states. Two residual variances for the new proposed segments are then constructed from the current single residual variance. Finally, two new vectors of linear parameters are sampled. If a death move is proposed, we have that $k^{\;p}=k^{\;c}-1$. Hence, a candidate change-point to be removed is selected from the vector of existing change-points, with probability $\frac{1}{k^{\;c}}$. The latter involves merging two existing partitions. The number of frequencies and their values in the proposed segments are selected from the current states. A single residual variance is constructed from the current variances relative to the segments affected by the relocation. Finally, a new vector of linear coefficient is drawn. For both type of moves, these updates are jointly accepted or rejected.

## 5 Simulation Studies

We carry out simulation studies to explore the performance of our method, which will be referred to as Automatic Nonstationary Oscillatory Modeling (AutoNOM). In Section 5.1, we illustrate the performance of our methodology when the simulated data are generated from the proposed model. Section 5.2 deals with scenarios when the model is misspecified relative to the generating process. Our results are compared with two state-of-the-art existing methods.

### 5.1 Illustrative Example

**Fig. 1:**
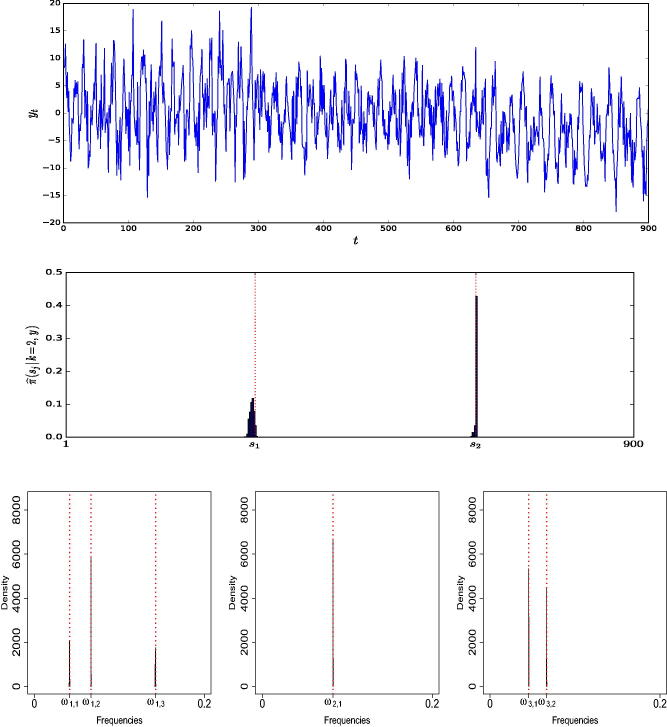
Illustrative example. (Top) Simulated time series. (Middle) Estimated posterior distribution for the location of the change-points, conditioned on *k* = 2. The dotted vertical lines represent true location of change-points. (Bottom) Estimated posterior distribution of the frequencies for each different segment, conditioned on *k* = 2, $m_{1}=3,m_{2}=1$, and $m_{3}=2$. The dotted vertical lines represent true values of the frequencies.

In this simulation example, we generate a time series consisting of *n* = 900 data points from model (1) with *k* = 2 change-points located at positions $s_{\;(2)}=(300,650)$, and fixed number of frequencies per regime $m_{\;(2)}=(3,1,2)$. (Further details of the parameterization are available in supplementary materials, Section 1.1.) Figure 1 (top panel) shows a realization from this model. The prior means $\lambda_{\omega }$ and *λ_s_*, say, on the number of frequencies and change-points, respectively, were set to 2, to reflect a fair degree of prior information on their numbers. We discuss in Section 1.2 of the supplementary materials that AutoNOM was relatively insensitive to these prior specifications, for this example. The maximum number of change-points $k_{\text{max}}$ was set to 15, and the maximum number of frequencies per regime $m_{\text{max}}$ was set to 10. Furthermore, we fixed $\psi_{s}=20$ and $\phi_{\omega }$ = 0.25 (Appendix A.1.2) for the uniform distribution for sampling the frequencies. The full estimation algorithm was ran for 20,000 updates, 5000 of which are discarded as burn-in period. The estimation took 390 sec with a (serial) program written in Julia 0.62 on a Intel® Core™ i7-4790S Processor 16 GB RAM. The results, summarized in Table 1 clearly show that a model with two change-points has the highest estimated posterior probability (left panel) and that AutoNOM correctly identifies the right number of significant frequencies in each regime (right panel).

**Table 1:** Illustrative example.

k	$\hat{\pi }\;(\;k\;|\;y)$	m	$\hat{\pi }\;(\;m_{1}\;|\;k=2,\;y)$	$\hat{\pi }\;(\;m_{2}\;|\;k=2,\;y)$	$\hat{\pi }\;(\;m_{3}\;|\;k=2,\;y)$
0	0.00	1	0.00	0.99	0.00
1	0.02	2	0.00	0.01	0.98
2	0.97	3	0.98	0.00	0.02
3	0.01	4	0.02	0.00	0.00
4	0.00	5	0.00	0.00	0.00

NOTES: (Left panel) posterior probabilities for number of change-points; (right panel) posterior probabilities for number of frequencies in each regime, conditioned on *k* = 2.

Figure 1 (middle panel) shows the estimated posterior distribution for the location of the change-points, conditioned on three segments. The posterior means of the change-point locations are $\hat{E}\;(s_{1}\;|\;k=2,\;y)=298.7$ and $\hat{E}\;(s_{2}\;|\;k=2,\;y)=650.1$. Figure 1 (bottom panel) shows that the estimated posterior distributions are an excellent match to the true frequencies. In addition, we provide details about acceptance rates in supplementary materials, Section 2.

#### 5.1.1 Detecting Spectral Peaks

We simulate a time series from the same simulation model as above with the only difference that the residual variances were set equal to one for all segments and thus are smaller than above. The performance of AutoNOM is compared with two existing methods, namely the Bayesian adaptive spectral estimation for nonstationary time series proposed by Rosen, Wood, and Stoffer (2012), referred to as AdaptSPEC, and the frequentist piecewise vector autoregressive method of Davis, Lee, and Rodriguez-Yam (2006), referred to as AutoPARM. Specifically, we explore the performances of these methodologies in identifying the number and location of change-points, and the number and location of frequency peaks in each estimated segment. AdaptSPEC requires the user to specify in advance the number of basis function *J* used for smoothing the periodogram in the segments. We run AdaptSPEC for two different specifications, namely *J* = 7 and *J* = 15 basis functions. The model is fitted with a total of 15,000 iterations, 5,000 of which are discarded as burn-in, by using the R package provided by the authors. Posterior samples of peak frequencies are obtained by considering the modes of the spectrum per MCMC iteration. AutoPARM is performed with default tuning parameters. We note that Davis, Lee, and Rodriguez-Yam (2006) do not discuss computation of confidence intervals for frequencies.

**Fig. 2:**
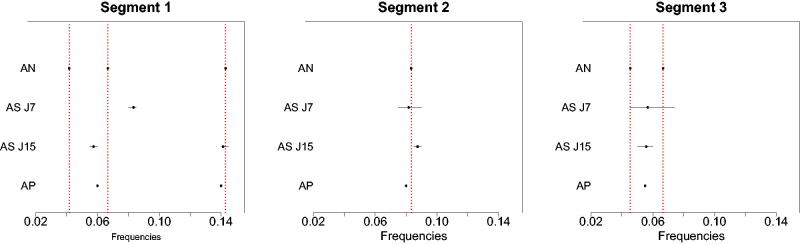
Illustrative example with unitary residual variances. Estimated frequency peaks for AutoNOM (AN), AdaptSPEC (AS, J=7,15), and AutoPARM (AP); 95% credible intervals (horizontal lines) are also reported for Bayesian methods. Dotted vertical lines are true locations of the frequency peaks.

The modal number of change-points for AdaptSPEC is 2 for both *J* = 7 and *J* = 15, with posterior probability $\hat{\pi }\;(k=2\;|\;y)$ of 76% and 88%, respectively; the modal number of change-points for AutoNOM is 2 and AutoPARM identifies 2 change-points as well. Conditioned on the modal number of change-points, Table 2 displays the estimated location of changes (left panel) and frequency peaks (right panel) for the different compared methods, where we report the standard deviation for the estimate obtained from the empirical distribution of the posterior samples. Similarly, we show in Figure 2 the estimated location of the frequency peaks and their 95% credible intervals, for each of the three identified segments; dotted vertical lines represents the true location of the frequency peaks. Results for AutoNOM are conditioned on the modal number of frequencies per regime.

**Table 2:** Illustrative example with unitary residual variances.

	*s*_1_	*s*_2_	$\omega_{1,1}$	$\omega_{1,2}$	$\omega_{1,3}$	$\omega_{2,1}$	$\omega_{3,1}$	$\omega_{3,2}$
True	300	650	0.042	0.067	0.143	0.083	0.046	0.067
AN	300.87	650.46	0.042	0.067	0.142	0.083	0.045	0.067
AN	(0.97)	(0.31)	(0.0004)	(0.0002)	(0.0005)	(0.0002)	(0.0003)	(0.0003)
AS J7	319.65	628.51	0.083	–	–	0.082	0.057	–
AS J7	(15.36)	(37.35)	(0.0030)	–	–	(0.0070)	(0.0010)	–
AS J15	298.5	647.01	0.057	0.141	–	0.088	0.056	–
AS J15	(1.21)	(0.14)	(0.0030)	(0.0020)	–	(0.0050)	(0.0030)	–
AP	299	648	0.060	0.140	–	0.080	0.055	–

NOTES: Estimated change-points locations (left panel) and frequency peaks (right panel) for AutoNOM (AN), AdaptSPEC (AS *J* = 7, 15), and AutoPARM (AP); posterior standard deviations are also reported for Bayesian methods.

It becomes clear that the detection of periodicities by AdaptSPEC is affected by the specification of the number of spline basis functions used for the smoothing, where increasing the number of basis function yields a better performance for AdaptSPEC. The example also shows that smoothing by splines may lead to peaks in the periodogram to be over-smoothed and neighboring close peaks to be merged. AutoPARM seems to also suffer from the latter problem.

When we increased the residual variance to the high levels set originally, AdaptSPEC failed to detect any change-points for both *J* = 7 and *J* = 15, with posterior probability $\hat{\pi }\;(k=0\;|\;y)$ of 69% and 93%, respectively, while AutoPARM found 7 change-points and thus severely overestimates their number. Our conclusion from this comparison is that although AdaptSPEC and AutoPARM may be well suited for time series processes with smooth time-varying spectra with few or no peaks, both methods are severely challenged in detecting changes in spectra that exhibit pronounced peakedness, possibly at nearby frequencies, as can be expected to occur in reality for the type of time series that we wish to analyze.

### 5.2 Misspecified Model

We investigate the performance of our proposed method for identifying spectral peaks when the model is misspecified relative to the generating process. In particular, we explored simulation studies under three different settings. In the first two scenarios we generated data from two types of autoregressive (AR) processes, namely a piecewise AR process and a slowly varying AR process. We compare the performance of our procedure with AutoPARM and AdaptSPEC. In the third setting, we assumed that the innovations are *t*-distributed, and therefore violate the Gaussianity assumption of $\varepsilon_{t}$ in Equation (3). For all models, our estimation algorithm was run for 20,000 iterations, 5000 of which were used as burn in, and the hyperparameters were chosen as $\phi_{s}=40,\lambda_{\omega }=0.05$ and $\lambda_{s}=0.01$.

#### 5.2.1 Piecewise Autoregressive Process

**Fig. 3:**
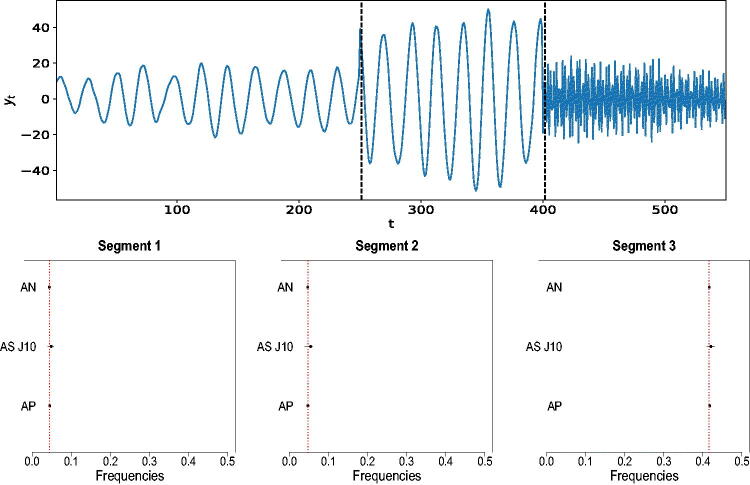
Piecewise AR process. (Top) A realization from model (11). Vertical dotted lines are the estimated locations of the change-points. (Bottom) Estimated frequency peaks for AutoNOM (AN), AdaptSPEC (AS *J* = 10), and AutoPARM (AP); 95% credible intervals (horizontal lines) are also reported for Bayesian methods. Dotted vertical lines are true locations of the frequency peaks.

As pointed out by a referee, although modeling a time series as a linear combination of a finite number of sinusoids plus noise is common in the signal processing literature, such line-spectrum based models are rare in the statistics literature. In fact, it is commonly assumed that the power spectrum is continuous across frequencies. We investigate the performance of the proposed procedure when analyzing data generated from a piecewise AR process whose local spectral density functions show sharp peaks. Specifically, a realization is simulated from

(11) $$y_{t}=\{\begin{array}{cc} 1.9\;y_{t-1}–0.975\;y_{t-2}+\varepsilon_{t}^{\left(1\right)} & \text{for}\;1\leq t\leq 250\\ 1.9\;y_{t-1}–0.991\;y_{t-2}+\varepsilon_{t}^{\left(2\right)} & \text{for}\;251\leq t\leq 400\\ -1.35\;y_{t-1}–0.37\;y_{t-2}+0.36\;y_{t-3}+\varepsilon_{t}^{\left(3\right)} & \text{for}\;401\leq t\leq 550, \end{array}$$

where $\varepsilon_{t}^{\left(1\right)}\overset{\text{iid}}{∼}N(0,0.25)$ and $\varepsilon_{t}^{\left(i\right)}\overset{\text{iid}}{∼}N(0,1)$ for *i* = 2, 3. Figure 3 (top panel) shows a realization from model (11). After applying our methodology AutoNOM, the posterior probability of two change-points is 97.93% and the posterior means of the change-point locations are $\hat{E}\;(s_{1}\;|\;k=2,\;y)=251.19$ and $\hat{E}\;(s_{2}\;|\;k=2,\;y)=401.56$. The estimated location of the frequency peaks for our proposed procedure in comparison to AdaptSPEC and AutoPARM and the true values are shown in Figure 3 (bottom panels). It is evident that the proposed and existing methodologies successfully identify the true location of the frequency peaks in each segment, with AdaptSPEC showing less precision.

#### 5.2.2 Slowly Varying Autoregressive Process

**Fig. 4:**
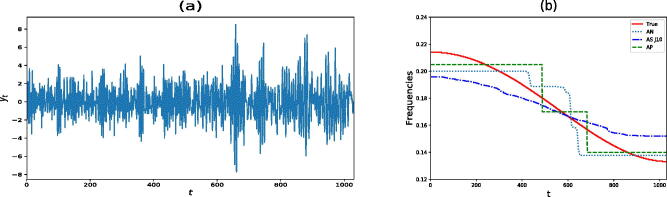
Slowly varying AR(2) process. (a) A realization from model (12). (b) True time varying frequency peak (solid line) and estimated time varying frequency peak for AutoNOM (AN), AdaptSPEC (AS *J* = 10), and AutoPARM (AP).

In this section, we analyze an AR process whose continuous spectral density is changing slowly over time. We note though that this scenario is a large departure from the assumptions of our model. In particular, we consider the same slowly varying AR(2) process investigated by Ombao et al. (2001) and Davis, Lee, and Rodriguez-Yam (2006), namely

(12) $$y_{t}=a_{t}\;y_{t-1}-0.81\;y_{t-2}+\varepsilon_{t},\;t=1,\ldots ,1031,$$

where $a_{t}=0.8\;[1-0.5\;\text{cos}\;\;(\pi t/1031)]$ and $\varepsilon_{t}\overset{\text{iid}}{∼}N(0,1)$. Notice that the parameter *a_t_* is changing gradually over time whereas the coefficient associated with the second lag remains constant. A realization from model (12) is shown in Figure 4(a) and the corresponding time varying frequency peak is displayed in Figure 4(b) as a solid line. Figure 4(b) also shows the estimated time varying frequency peak for AutoNOM, AdaptSPEC, and AutoPARM. For AutoNOM and AdaptSpec, the time changing frequency peak has been averaged across the MCMC samples, giving a smoother estimate (especially for AdaptSPEC) than the one obtained by AutoPARM. For each method, we compute the residual sum of squares $\text{RSS}=\sum_{t=1}^{1031}{\left(\omega_{t}-{\hat{\omega }}_{t}\right)}^{2}$ between the true time changing frequency peak *ω_t_* and its estimate ${\hat{\omega }}_{t}$. The RSS in this example was 0.111, 0.174, and 0.085 for AutoNOM, AdaptSPEC, and AutoPARM, respectively. It is clear that even in this scenario where the data generating model was very different from the underlying assumptions of our model, our approach seems to outperform AdaptSPEC and remains competitive with AutoPARM in estimating the time varying frequency peak.

#### 5.2.3 Non-Gaussian Time Series

**Fig. 5:**
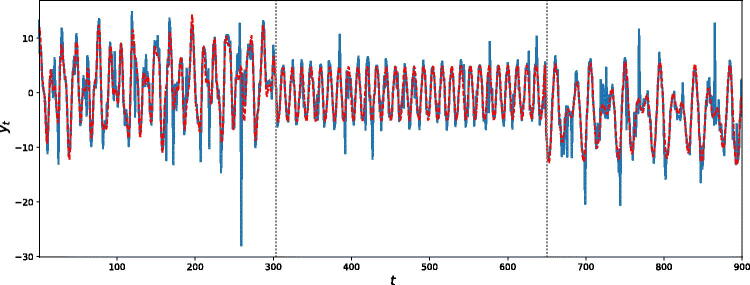
Illustrative example with *t*-distributed residual variances. Simulated time series (solid line) and estimated signal (dotted line). The dotted vertical lines represent the estimated location of the change-points.

We investigate the performance of our approach in the scenario when the innovations are *t*-distributed. We simulate a time series from the same simulation model presented in Section 5.1, where errors were generated from a *t*-distribution with 2, 3, and 2 degrees of freedom for the sequence of three segments, respectively. The degrees of freedom were chosen low such that the corresponding distributions show heavy tails. A realization of this time series is shown in Figure 5. Our proposed methodology correctly identifies the 2 change-points, as the estimated posterior probability $\hat{\pi }\;(k=2\;|\;y)$ is 0.99. The posterior means of the change-point locations are $\hat{E}\;(s_{1}\;|\;k=2,\;y)=303.6$ and $\hat{E}\;(s_{2}\;|\;k=2,\;y)=650.5$, showing an excellent match to the true values $s_{\;(2)}=(300,650)$. Furthermore, the posterior mode of the number of frequencies in each segment is ${\hat{m}}_{\;(2)}=(3,1,2)$, which is a correct estimate of $m_{\;(2)}=(3,1,2)$. We also display in Figure 5 the estimated signal (using Equation (1), supplementary materials) as a dotted line. We can conclude that, although our model assumes Gaussianity, AutoNOM seems to perform well even in the case where the oscillatory underlying process is *t*-distributed with heavy tails.

## 6 Case Studies

The development of our methodology was motivated by the following two case studies where dense physiological signals were observed which exhibit unknown periodicities whose role may change over time in a more or less abrupt manner and where their detection is of relevance to the health and well-being of the subject.

### 6.1 Analysis of Human Skin Temperature

**Fig. 6:**
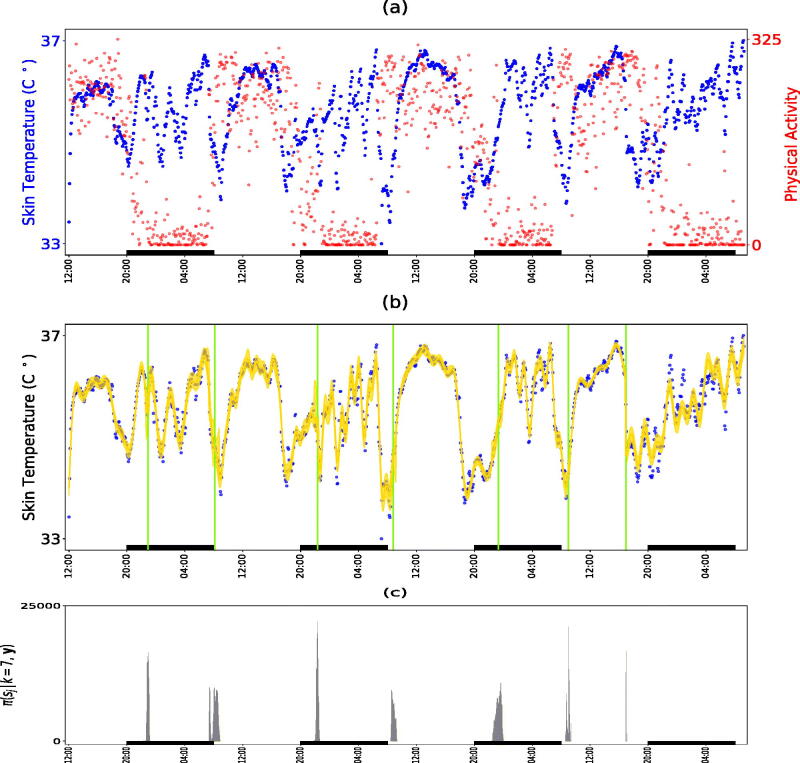
Analysis of skin temperature of a healthy subject. Panel (a) are the time series of skin temperature and corresponding physical activity. Panel (b) is the estimated signal (solid line) along with its 95% credible interval; vertical lines are the estimated locations of the change-points. Panel (c) is the estimated posterior density histogram of the locations of the changes, conditioned on k=7 change-points. Rectangles on the time axis of each plot correspond to periods from 20.00 to 8.00. The variation in skin temperature finds analogies with the rest-activity pattern that alternates between day activity and night rest.

The development of information and communication technologies, in particular widespread internet access and availability of mobile phones and tablets, allows considering new developments in the health care system. To address the issue of personalized medical treatment according to the circadian timing system of the patient, referred to as *chronotherapy* (Levi and Schibler 2007), a novel and validated noninvasive mobile e-Health platform pioneered by the French project PiCADo (Komarzynski et al. 2018) is used to record and teletransmit skin surface temperature as well as physical activity data (Huang et al. 2018) from an upper chest e-sensor. Figure 6(a) shows an example of 4 days of 5-min temperature recording for a healthy individual. The circadian rhythms in core and skin surface temperature are usually 8–12 hr out of phase, with respective maxima occurring near 16:00 at day time, and near 2:00 at night (Krauchi and Wirz-Justice 1994). The early night drop in core body temperature, which is critical for triggering the onset of sleep (Van Someren 2006), results from the vasodilatation of the skin vessels and associated rise in skin surface temperature (Kräuchi 2002). Under the assumption of stationarity, Komarzynski et al. (2018) analyzed the skin temperature time series identifying both strong 12 hr (circahemidian) and 24 hr (circadian) rhythms.

**Fig. 7:**
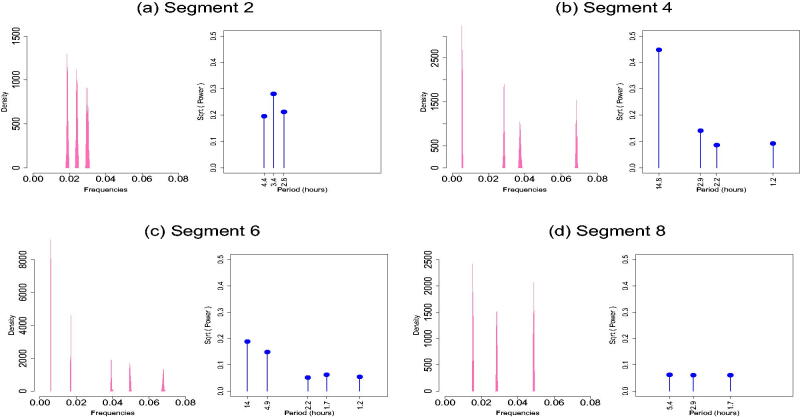
Spectral properties for segments corresponding to night rest. Estimated posterior distribution of the frequencies along with square root of the estimated power of the corresponding frequencies. The results are conditional on the modal number of frequencies per segment.

Here, we applied our methodology to the skin-temperature time series shown in Figure 6(a) for 300,000 iterations, discarding the first 100,000 updates as burn-in. The maximum number of change-points $k_{\text{max}}$ was set to 10, whereas the maximum number of frequencies per regime $m_{\text{max}}$ was set to 5. The estimated number of change-points had a mode at 7, with $\hat{\pi }\;(\;k=7\;|\;y)=0.97$ and their estimated posterior distributions are shown in Figure 6(c). Inspecting them alongside the physical activity data we can see that the change points mainly correspond to the start and endpoints of the prolonged rest periods at nights showing that skin temperature alternates between day activity and night rest including sleep. Figure 7 shows the estimated posterior distribution of the frequencies for the sleep segments (2, 4, 6, 8) along with the square root of the estimated power of the corresponding frequencies, where the power of each is frequency $\omega_{j,l}$ is summarized by the sum of squares of the corresponding linear coefficients, that is, $I\;(\omega_{j,l})\;=\beta_{j,l}^{\;{\left(1\right)}^{\;\;2}}+\beta_{j,l}^{\;{\left(2\right)}^{\;\;2}}$ (Shumway and Stoffer 2005). Figure 6(b) shows the piecewise fitted signal, along with a 95% credible interval obtained from the 2.5 and 97.5 empirical percentiles of the posterior sample using Equation (1), supplementary materials. Cycles of approximately 3 hr appear in segments 2, 4, and 6; cycles that range approximately 1–1.5 hr appear in segments 2, 4, 8 and cycles of around 2 hr appear in segments 4 and 6 while some longer periods identified in segments 4, 6, 8 indicate the presence of a trend.

Stages of sleep are characterized by ultradian oscillations between rapid eye movements (REM) and non-REM. The biological functionality that regulates the alternations between these two types of sleep is not yet much understood (Altevogt and Colten 2006). However, several physiological changes that occur over night differ between REM and non-REM phases, such as heart rate, brain activity, muscle tone and body temperature (Berlad et al. 1993; Pace-Schott and Hobson 2002). The body cycles between REM and non-REM sleep stages with an average length that ranges approximately between 70 and 120 min, and there are usually four to six of these sleep cycles each night (Carskadon and Dement 2005; Shneerson 2009). Our analysis was able to use skin temperature data alone to detect periods of sleep throughout the day and identify oscillatory behavior during the night, whose frequencies are compatible with ultradian oscillations between REM and different non-REM sleep stages.

A comparison with the current state-of-the-art methods, AutoPARM and AdaptSPEC, is provided in the supplementary materials, Section 4.1. Circadian and ultradian rhythmicity are expected because body temperature is known to be a circadian biomarker (Krauchi and Wirz-Justice 1994), but these existing methods fail. Furthermore, we notice that in the framework of analyzing circadian biomarker data, such as body temperature, a change in acrophase may be of interest to the clinician as this may be indicative of a disruption of the bodyclock. The methodology can indeed be used to investigate phase which can be computed from the sinusoidal function that characterizes the *j*th segment (see supplementary materials, Section 3).

### 6.2 Characterizing Instances of Sleep Apnea in Rodents

Sleep apnea is the temporary (≥ 2 breaths) interruption of breathing during sleep. Moderate or severe (≥ 15 events per hour) sleep apnea, occurs in about 50% of men and 25% of women over the age of 40 (Heinzer et al. 2015), with 91% of people with sleep apnea being undiagnosed (Tan et al. 2016). Sleep apnea is linked to many diseases. Patients with sleep apnea are at increased risk of: cardiovascular events (Lanfranchi et al. 1999), cancer (Nieto et al. 2012), liver disease (Sundaram et al. 2016), diabetes (Harsch et al. 2004), metabolic syndrome (Parish, Adam, and Facchiano 2007), cognitive decline (Osorio et al. 2016), and increased risk of dementia in the elderly (Lal, Strange, and Bachman 2012). The motivation of this research is to provide a statistical methodology that can be applied to analyze large breathing datasets resulting from in vivo plethysmograph studies in rats to characterize the occurrence of sleep apnea under different experimental conditions. If this could be attained, a concrete aid to the understanding of the pathological implications of this status could be provided to clinicians and experimental biologists.

An unrestrained whole-body plethysmograph is used to produce a breathing trace from freely behaving rats for periods of up to 3 hr. Plethysmographs were made using an 2 L air-tight box connected to a pressure transducer, with an air pump and outlet valve producing a flow rate of 2 L/min. Airflow pressure signals were amplified using Neurolog system (Digitimer) connected to a 1401 interface and acquired on a computer using *Spike2* software (CED).

**Fig. 8:**
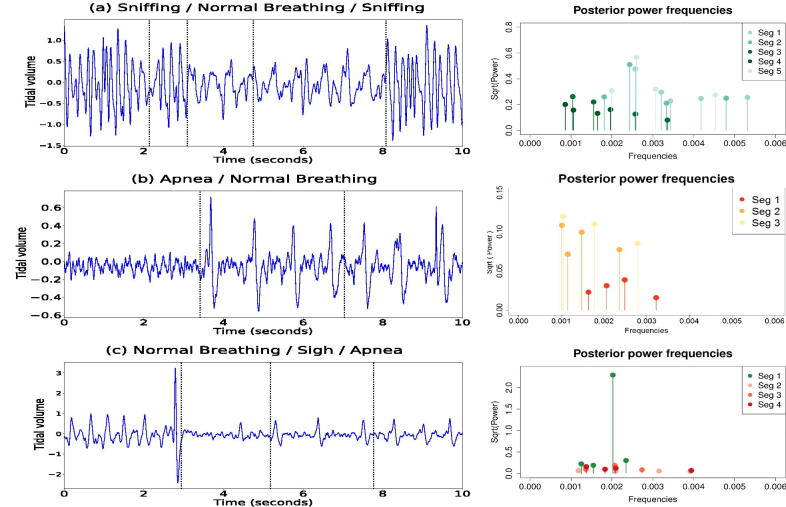
Plots of the respiratory traces of a rat (left panels) and corresponding estimated posterior power (right panels). Panel (a) is characterized by an alternation of sniffing and normal breathing. Panel (b) is a plot of the trace of a spontaneous apnea, followed by normal breathing. Panel (c) shows normal breathing followed by a sigh, and a post-sigh apnea. Dotted vertical lines correspond to the estimated locations of the change-points.

Apneas are subclassified as post-sigh apneas, if the preceding breath was at least 25% above the average amplitude of prior breaths, or spontaneous apneas, if there was no manifestation of a previous sigh (Davis and O’Donnell 2013). Airflow traces from the plethysmograph are shown in Figure 8 (left panels) and consist of three time series, which will be referred to as (a), (b), and (c). They correspond to different actions for this rat: (a) an alternation of sniffing and normal breathing; (b) spontaneous apnea followed by normal breathing; (c) normal breathing followed by a sigh, and a post-sigh apnea. We note that these actions were classified by eye by an experienced experimental researcher. Each time series contains 20,000 observations where the signal was sampled at 2000 Hz so that we have 2000 observations per second.

Our procedure allows us to set an upper bound, $\phi_{\omega }$, (Appendix A.1.2) for the uniform interval where the new frequencies are sampled. As the periodogram ordinates for these data were approximately zero for all frequencies larger than 0.01, we decided accordingly to set $\phi_{\omega }=0.01$. The locations of the changes (vertical lines) are displayed in Figure 8 (left panels). The posterior power of the frequencies, for each time series, is shown in Figure 8 (right panels). These results are conditional on the modal number of change-points and the modal number of frequencies per segment. For each dataset, we summarize in Table 3 the spectral properties of each partition by displaying the periodicities corresponding to the first two largest values of the estimated power. When the rat is sniffing, (a), the air flow trace oscillates with a dominant period of approximately 0.2 sec, namely 5 cycles per second. Normal breathing, (a) and (b), is characterized by lower frequencies and lower magnitude than sniffing, by oscillating with a dominant period of around 0.5 sec, namely around 2 cycles every second. Apneas, (b) and (c), appear to be characterized by higher frequencies than normal breathing but with a lower power, with dominant periods of around 0.25 and 0.35 sec. Notice that in the first partition of (c), the highest value of the power corresponds to the frequency responsible for a sigh before apnea. Moreover, our methodology identifies different frequencies that explain the variation between the third and fourth partition of (c), leading to the hypothesis that there might be a time changing spectrum during the occurrence of an apnea instance. A comparison of our results with the results from AutoPARM and AdaptSPEC is provided in the supplementary materials, Section 4.2.

**Table 3:** Spectral properties of respiratory traces of a rat.

	(a)		(b)		(c)	
	Period	Power	Period	Power	Period	Power
Segment 1	0.19	0.2272	0.20	0.0015	0.25	5.2540
	0.15	0.0883	0.25	0.0010	0.21	0.0915
Segment 2	0.20	0.2613	0.49	0.0117	0.27	0.0107
	0.28	0.0686	0.34	0.0099	0.42	0.0043
Segment 3	0.48	0.0692	0.48	0.0145	0.24	0.0365
	0.32	0.0491	0.28	0.0122	0.37	0.0095
Segment 4	0.58	0.0400	–	–	0.36	0.0253
	0.47	0.0251			0.24	0.0155
Segment 5	0.19	0.3231	–	–	–	–
	0.16	0.1044				

NOTES: Periodicities (in seconds) corresponding to the first two largest values of the estimated power, for each time series (a), (b), and (c).

## 7 Summary and Discussion

We developed a novel Bayesian methodology for analyzing nonstationary time series that exhibit oscillatory behavior. Our approach is based on the assumption that, conditional on the position and number of change-points, the time series can be approximated by a piecewise changing sinusoidal regression model. The timing and number of changes are unknown, along with the number and values of relevant periodicities in each regime. Bayesian inference is performed via a reversible jump MCMC algorithm that can simultaneously estimate both the number and location of change-points, as well as the number, frequency and magnitude of sinusoids within each segment. Our methodology can be seen as a novel and relevant extension of the work in Andrieu and Doucet (1999) to the nonstationary setting.

We illustrated the utility of our methodology in two case studies. First, we analyzed human skin temperature time series data obtained from a wearable device, which exhibited unknown periodicities that changed over time in an abrupt manner. Our proposed methodology identified interesting oscillations whose frequencies are consistent with ultradian oscillations between REM and non-REM sleep stages. Second, we characterized the occurrence of sleep apnea in large breathing datasets resulting from in vivo plethysmograph studies on rodents. Our spectral investigation was able to distinguish very sharp peaks, corresponding to different nearby frequencies, that are responsible for the different actions of the rodent.

Although we have not discussed this in detail here, several diagnostics for monitoring convergence were carried out in both simulation and case studies. In particular, we verified that the target posterior distribution reached a stable regime by analyzing the trace plot of the log-likelihood across MCMC iterations (Marin and Robert 2007). We are aware that assessing convergence only based on this simple tool may sometimes be misleading since stable values of the log-likelihood could simply mean that the Markov chain is stuck in some local mode of the posterior distribution. Additionally, conditioned on the modal number of change-points and modal number of frequencies per regime, we have also monitored (within-model) convergence by analyzing the traces and running averages plots for all parameters across MCMC iterations, with satisfactory results. Comparable results were also obtained when running several chains starting at over-dispersed initial values. We notice that the diagnostic tool used by Bruce et al. (2018) and Li and Krafty (2019) to assess convergence for reversible jump MCMC samplers appears relevant. In the context of adaptive spectral analysis of nonstationary time series, they point out that although the number of partitions change across models, a power spectrum is defined at each time point. The power spectrum is modeled with a fixed number of splines, yielding a vector of summary measures of parameters that maintain the same interpretation across models in their samplers. However, our proposed sampler has a further layer of variable dimensionality, as not only the number and locations of the change-points may change from one iteration to the next, but also the number of frequencies in each segment are not fixed throughout the simulation.

We conclude this article by noticing that, although a Gaussian distribution is assumed, it is conceivable that our model can be extended to allow for other error distributions in Equation (1). For example, a generalized linear model (McCullagh and Nelder 1989) may be used to model periodic count data by assuming that the observed data follows a Poisson distribution, that is, $y_{t}∼\text{Poisson}\;(\mu_{t})$. The logarithmic link function of the expected value *μ_t_* of the response variable *y_t_* may be expressed as $\text{log}\;\;(\;\mu_{t}\;)=\sum_{j=1}^{k+1}f\;(t,\;\beta_{\;j},\;\omega_{\;j}\;)1_{\left[\;t\;\in \;I_{j}\;\right]}$, where the definitions of the variables are the same as for Equation (1). Bayesian inference can, in principle, be achieved in a similar way as described in the article, namely by iterating between segment and change-point model moves, where the formulation of the acceptance probabilities and some proposal distributions need to be modified accordingly. We believe that such an extension would find use in several ranges of applications, for example, in studying population cycles in ecology and epidemiology, where the abundance of species are measured as count variables (White and Bennetts 1996; Bhaskaran et al. 2013; Bramness et al. 2015).

## Supplementary Material

Supplemental Material

## Supplementary Materials

Supplementary materials are available and include further details about simulation studies, acceptance rates, phase investigation, and performances of existing methods in the case studies. Code that implements the methodology and the data used in the case studies are available as online supplemental material and can be also found at https://github.com/Beniamino92/AutoNOM.

## Appendix A: Details of the Sampling Scheme

### A.1 Updating the Segment Model

#### A.1.1 Within-Model Move

**Sampling** ω: To obtain samples from the conditional posterior distribution $\pi \;(\omega \;|\;\beta ,\;\sigma^{2},\;m,\;y)$ (see Equation (8)), we draw the frequencies one-at-time using a mixture of M-H steps. To explore the parameter space efficiently, we design a mixture distribution $q\;(\;\omega_{l}^{\;p}\;|\;\omega_{l}^{\;c}\;)$, so that

(A.1) $$\begin{array}{c} q\;(\;\omega_{l}^{\;p}\;|\;\omega_{l}^{\;c}\;)=\delta_{\omega }\;q_{1}\;(\;\omega_{l}^{\;p}\;|\;\omega_{l}^{\;c}\;)\\ \;+(1-\delta_{\omega })\;q_{2}\;(\;\omega_{l}^{\;p}\;|\;\omega_{l}^{\;c}\;),\;l=1,\ldots ,m, \end{array}$$

where *q*_1_ is defined in Equation (A.2), *q*_2_ is the density of a univariate normal $N\;(\omega_{l}^{\;c},\sigma_{\omega }^{2}),\delta_{\omega }$ is a positive real number such that $0\leq \delta_{\omega }\leq 1$, and *c* and *p* refer to current and proposed values, respectively. According to Equation (A.1) we carry out with probability $\delta_{\omega }$ a M-H step with proposal distribution $q_{1}\;(\;\omega_{l}^{\;p}\;|\;\omega_{l}^{\;c}\;)$,

(A.2) $$q_{1}\;(\;\omega_{l}^{\;p}\;|\;\omega_{l}^{\;c}\;)\propto \sum_{h\;=\;0}^{\tilde{n}-1}I_{h}\;1_{[\;h/n\;\;\leq \;\;\omega_{l}^{\;\;p}\;<\;\;(h+1)/n\;]\;},$$

where $\tilde{n}=\left\lfloor n/2\right\rfloor$ and *I_h_* is the value of the squared modulus of the discrete Fourier transform of the observations ***y*** evaluated at frequency *h*/*n*

$$I_{h}={\left|\;\sum_{j\;=\;a}^{b}y_{j}\;\;\text{exp}\;\left(-i\;2\pi \;\frac{h}{n}\;\right)\;\right|}^{\;2}.$$

In this way frequencies are proposed from regions in parameter space with high posterior density, yielding a Markov chain which converges quickly to its invariant distribution. The proposal distribution $q_{1}\;(\;\omega_{l}^{\;p}\;|\;\omega_{l}^{\;c}\;)$ is independent of the current state $\omega_{l}^{\;c}$. The acceptance probability for this move is

$$\alpha =\min\left\{1,\frac{\pi \;(\omega^{\;p}\;|\;\beta ,\;\sigma^{2},\;m,\;y)}{\pi \;(\omega^{\;c}\;|\;\beta ,\;\sigma^{2},\;m,\;y)}\times \frac{q_{1}\;(\;\omega_{l}^{\;c}\;)}{q_{1}\;(\;\omega_{l}^{\;p}\;)}\right\},$$

where $\omega^{\;p}=(\omega_{1}^{\;c},\ldots ,\omega_{l-1}^{\;c},\omega_{l}^{\;p},\omega_{l+1}^{\;c},\ldots ,\omega_{m}^{\;c})\prime$. On the other hand, with probability $1-\delta_{\omega }$, we perform a random walk M-H step with proposal distribution $q_{2}\;(\;\omega_{l}^{\;p}\;|\;\omega_{l}^{\;c}\;)$, whose density is a univariate normal distribution with mean $\omega_{l}^{\;c}$ and variance $\sigma_{\omega }^{2}$, that is, $\omega_{l}^{\;p}\;|\;\omega_{l}^{\;c}\;∼N(\;\omega_{l}^{\;c},\;\sigma_{\omega }^{2}\;)$. This perturbation around the current value $\omega_{l}^{\;c}$ allows a local exploration of the conditional posterior distribution. The acceptance probability for this move is

$$\alpha =\min\left\{1,\frac{\pi \;(\omega^{\;p}\;|\;\beta ,\;\sigma^{2},\;m,\;y)}{\pi \;(\omega^{\;c}\;|\;\beta ,\;\sigma^{2},\;m,\;y)}\right\}.$$

Setting $\delta_{\omega }$ to a relative low value integrates a fairly high acceptance rate with a quick exploration of the parameter space. For our experiments, we set $\sigma_{\omega }^{2}={\left(1/50n\right)}^{2}$ and $\delta_{\omega }=0.2$.

**Sampling** β**:** Given values of ω and $\sigma^{2}$, the vector of linear parameters β can be sampled via a M-H step from the target posterior conditional distribution $\pi \;(\;\beta \;|\;\omega ,\;\sigma^{2},\;m,\;y)$ (see Equation (9)). The proposed vector of coefficients $\beta^{\;p}$ can be drawn from normal approximations to their posterior conditional distribution (e.g., Gilks, Richardson, and Spiegelhalter 1995; Rosen, Wood, and Stoffer 2012),

(A.3) $$\beta^{\;p}∼N_{2m+2}\;(\;{\hat{\beta }}^{\;p},\;{\hat{\Sigma }}^{\;p}),$$

where

$${\hat{\beta }}^{\;p}=\underset{\beta^{\;\;p}}{\text{arg}\;\text{max}}\;\pi \;(\;\beta^{\;p}\;|\;\omega ,\;\sigma^{2},\;m,\;y),$$

and

$${\hat{\Sigma }}^{\;p}={\left\{-\frac{\partial^{2}\;\text{log}\;\;\pi \;(\;\beta^{\;p}\;|\;\omega ,\;\sigma^{2},\;m,\;y)}{\partial \beta^{\;p}\;\partial \beta^{\;p\;\prime }}{|}_{\beta^{\;\;p}\;=\;{\hat{\beta }}^{\;\;p}}\right\}}^{-1}.$$

The proposal for $\beta^{\;p}$ is independent of the current values $\beta^{\;c}$, and the acceptance probability for this move is

$$\alpha =\min\left\{1,\frac{\pi \;(\;\beta^{\;p}\;|\;\omega ,\;\sigma^{2},\;m,\;y)}{\pi \;(\;\beta^{\;c}\;|\;\omega ,\;\sigma^{2},\;m,\;y)}\times \frac{q\;(\;\beta^{\;c}\;)}{q\;(\;\beta^{\;p}\;)}\right\},$$

where $\;q\;(\;\beta^{\;c}\;)$ and $q\;(\;\beta^{\;p}\;)$ denote the normal proposal densities $N_{2m+2}\;(\;{\hat{\beta }}^{\;c},\;{\hat{\Sigma }}^{\;c})$ and $N_{2m+2}\;(\;{\hat{\beta }}^{\;p},\;{\hat{\Sigma }}^{\;p})$, respectively.

#### A.1.2 Between-Model Moves

The number of frequencies on a segment is proposed to either increase or decrease by one. Let $\theta^{\;c}=(\;\beta^{\;c\;\prime },\;\omega^{\;c\;\prime },\;\sigma^{\;2c}\;)\prime$ and assume the Markov chain is currently at $\left(\;m^{\;c},\;\theta^{\;c}\;\right)$. We propose a move to $\left(\;m^{\;p},\;\theta^{\;p}\;\right)$ by drawing from a proposal density $q\;(\;m^{\;p},\;\theta^{\;p}\;|\;m^{\;c},\;\theta^{\;c}\;)$ and accepting this update with probability

(A.4) $$\begin{array}{c} \alpha =\min\{1,\frac{L(\;m^{\;p},\;\theta^{\;p}\;|\;y)}{L(\;m^{\;c},\;\theta^{\;c}\;|\;y)}\times \frac{\pi (m^{\;p})\;\pi (\theta^{\;p}\;|\;m^{\;p})}{\pi (m^{\;c})\;\pi (\theta^{\;c}\;|\;m^{\;c})}\\ \;\times \frac{q\;(m^{\;c},\;\theta^{\;c}\;|\;m^{\;p},\;\theta^{\;p}\;)}{q\;(m^{\;p},\;\theta^{\;p}\;|\;m^{\;c},\;\theta^{\;c}\;)}\}. \end{array}$$

The proposed state $\left(\;m^{\;p},\;\theta^{\;p}\;\right)$ is drawn by first drawing $m^{\;p}$, followed by $\omega^{\;p}$, $\beta^{\;p}$ and $\sigma^{\;2p}$. In fact, we can rewrite the proposal density as

$$\begin{array}{c} q\;(m^{\;p},\;\theta^{\;p}\;|\;m^{\;c},\;\theta^{\;c}\;)=q\;(m^{\;p}\;|\;m^{\;c})\times q\;(\theta^{\;p}\;|\;m^{\;p},\;m^{\;c},\;\theta^{\;c})\\ =q\;(m^{\;p}\;|\;m^{\;c})\times q\;(\omega^{\;p}\;|\;m^{\;p},\;m^{\;c},\;\theta^{\;c})\\ \;\times q\;(\;\beta^{\;p}\;|\;\omega^{\;p},\;m^{\;p},\;m^{\;c},\;\theta^{\;c})\\ \;\times q\;(\;\sigma^{\;2p}\;\;|\;\;\beta^{\;p},\;\omega^{\;p},\;m^{\;p},\;m^{\;c},\;\theta^{\;c}). \end{array}$$

**Birth move:** If a birth move is proposed, we have that $m^{\;p}=m^{\;c}+1$. The proposed frequency vector $\omega^{\;p}$ is constructed as

$$\omega^{\;p}=(\omega_{1}^{c},\ldots ,\;\omega_{m^{c}}^{c},\;\omega_{m^{p}}^{\;p})\prime ,$$

namely by keeping the current vector of frequencies and proposing an additional frequency $\omega_{m^{p}}^{\;p}$. Alternatively to Andrieu and Doucet (1999), we choose to sample $\omega_{m^{p}}^{\;p}$ uniformly on the interval $\left(0,\;\phi_{\omega }\right)$, where $0<\phi_{\omega }<0.5$. The value of $\phi_{\omega }$ can be chosen to reflect prior information about the significant frequencies that drive the variation in the data, for example, by choosing $\phi_{\omega }$ in the low frequencies range ($0<\phi_{\omega }<0.1$). Additionally, for computational and/or modelling reasons, we would like not to sample frequencies that are too close to each other. Hence, we choose to draw a candidate value $\omega_{m^{p}}^{p}$ uniformly from the union of intervals of the form $\left[\omega_{l}^{c}+\psi_{\omega },\;\omega_{l+1}^{c}-\psi_{\omega }\right]$, for $l=0,\ldots ,m_{c}$ and denoting $\omega_{0}^{\;c}=0$ and $\omega_{\;m^{c}+1}^{c}=\phi_{\omega }$. Here, $\psi_{\omega }$ is a fixed minimum distance between frequencies, which is chosen larger than $\frac{1}{n}$; in fact, when the separation of two frequencies is less than the *Nyquist step* (Priestley 1981), that is, $|\;\omega_{\;l}-\omega_{\;l+1}\;|<\frac{1}{n}$, the two frequencies are indistinguishable (Dou and Hodgson 1995). Moreover, we sort the proposed vector of frequencies $\omega^{\;p}$ to ensure identifiability and perform practical estimation, as suggested in Andrieu and Doucet (1999). For proposed $\omega^{\;p}$ and given $\sigma^{\;2c}$, the proposed vector of linear coefficients $\beta^{\;p}$ is sampled following the same procedure of Section A.1.1, namely we draw $\beta^{\;p}$ from a normal approximation to their posterior conditional distribution $\pi \;(\;\beta^{\;p}\;|\;\omega^{\;p},\;\sigma^{\;2c},\;m^{\;p},\;y\;)$. Finally, the residual variance $\sigma^{\;2p}$ is sampled directly from its posterior conditional distribution $\pi \;(\sigma^{\;2p}\;|\;\omega^{\;p},\;\beta^{\;p},\;m^{\;p},\;y)$ (see Equation (10)). The proposed state $\left(m^{p},\;\theta^{\;p}\;\right)$ is accepted or reject in a M-H step with probability

$$\begin{array}{c} \alpha =\min\{1,\;\frac{L(\;\theta^{\;p},\;m^{\;p}\;|\;y)}{L(\;\theta^{\;c},\;m^{\;c}\;|\;y)}\times \frac{\pi \;(m^{\;p})\;\pi \;(\theta^{\;p}\;|\;m^{\;p})}{\pi \;(m^{\;c})\;\pi \;(\theta^{\;c}\;|\;m^{\;c})}\\ \;\times \frac{d_{m^{\;\;p}}\cdot (\frac{1}{m^{\;p}})\cdot q\;(\;\beta^{\;c}\;)\cdot q\;(\;\sigma^{\;2c}\;)}{b_{m^{\;\;c}}\cdot q\;(\omega_{m^{\;\;p}}^{\;p})\cdot q\;(\;\beta^{\;p}\;)\cdot q\;(\;\sigma^{\;2p}\;)}\}, \end{array}$$

where the likelihood function is given in Equation (5), π( m ) is the density of the Poisson distribution truncated at $m_{\text{max}},b_{m^{\;\;c}}$ and $d_{m^{\;\;p}}$ are defined in Equation (7), $q\;(\omega_{m^{p}}^{p})$ is the density of the uniform proposal for sampling the additional frequency, $q\;(\beta^{\;c})$ and $q\;(\beta^{\;p})$ are the multivariate normal proposal densities $N_{2m^{\;\;c}+2}\;(\;{\hat{\beta }}^{\;c},\;{\hat{\Sigma }}^{\;c})$ and $N_{2m^{\;\;p}+2}\;(\;{\hat{\beta }}^{\;p},\;{\hat{\Sigma }}^{\;p})$, respectively; $q\;(\;\sigma_{p}^{2}\;)$ and $q\;(\;\sigma_{c}^{2}\;)$ are the Inverse-Gamma proposal densities defined in Equation (10).

**Death move:** If a death move is proposed, then $m^{\;p}=m^{\;c}-1$. A vector of frequencies $\omega^{\;p}$ is constructed by randomly selecting with probability $\frac{1}{m^{\;c}}$ one of the current frequencies as the candidate frequency for removal. Given $\omega^{\;p}$ and $\sigma^{\;2c}$, a vector of linear coefficients $\beta^{\;p}$ is sampled from a normal approximation to its posterior conditional distribution. Finally, conditioned on $\omega^{\;p}$ and $\beta^{\;p}$, the residual variance $\sigma^{\;2p}$ is drawn from its posterior Inverse-Gamma distribution. It is straightforward to see that the acceptance probability for the death step has the same form as the birth step, with the proper change of labelling of the variables, and the ratio terms inverted.

### A.2 Updating the Change-Point Model

#### A.2.1 Within-Model Move

Let $s_{\;(k)}^{\;c}=(s_{1}^{\;c},\ldots ,s_{k}^{\;c})\prime$ be the current vector of change-points locations, $m_{\;(k)}^{\;c}=(m_{1}^{\;c},\ldots ,m_{k+1}^{\;c})\prime$ be the current vector of number of frequencies, $\omega_{\;(k)}^{\;c}=(\omega_{1}^{\;c\;\prime },\ldots ,\omega_{k+1}^{\;c\;\prime })\prime$ be the current vector of frequencies. Let $\beta_{\;(k)}^{\;c}=(\;\beta_{1}^{\;c\;\prime },\ldots ,\beta_{k+1}^{\;c\;\prime })\prime$ and $\sigma_{\left(k\right)}^{\;2}=(\;\sigma_{1}^{\;2c},\ldots ,\sigma_{\;k+1}^{\;2c})\prime$ be the current vectors of linear coefficients and residual variances, respectively.

Let us also define $\theta_{\;(k)}^{\;c}=(\beta_{\;(k)}^{\;c\;\prime },\;\omega_{\;(k)}^{\;c\;\prime },\;\sigma_{\left(k\right)}^{\;2c\;\prime })\prime .$ Following Green (1995), a change-point, $s_{j}^{\;c}$ say, is randomly selected with probability $\frac{1}{k}$ from the existing set of change-points. To explore the parameter space in an efficient way and similar to above we construct a mixture distribution $q\;(\;s_{j}^{\;p}\;|\;s_{j}^{\;c}\;)$, as

(A.5) $$q\;(\;s_{j}^{\;p}\;|\;s_{j}^{\;c}\;)=\delta_{s}\;q_{1}\;(\;s_{j}^{\;p}\;|\;s_{j}^{\;c}\;)+(1-\delta_{s})\;q_{2}\;(\;s_{j}^{\;p}\;|\;s_{j}^{\;c}\;),$$

where *q*_1_ is the density of a Uniform $\left[s_{j-1}^{\;c}+\psi ,s_{j+1}^{\;c}-\psi \right]$, *q*_2_ is the density of a univariate normal $N\;(s_{j}^{\;c},\sigma_{s}^{2})$ and *δ_s_* is a positive real number such that $0\leq \delta_{s}\leq 1$. We propose with probability *δ_s_* a candidate value $s_{j}^{\;p}$ from the above uniform distribution where *ψ* is a fixed minimum time between change-points avoiding change-points being too close to each other. On the other hand, with probability $(1-\delta_{s}),s_{j}^{\;p}$ arises as a normal random walk proposal centered at the current change-point $s_{j}^{\;c}$. The proposed vector of change-points locations is denoted by

$$s_{\;(k)}^{\;p}=(s_{1}^{\;c},\ldots ,s_{j-1}^{\;c},s_{j}^{\;p},s_{j+1}^{\;c},\ldots ,s_{k}^{\;c})\prime ,$$

and hence the proposed value $s_{j}^{\;p}$ induces a new proposed data partition on $\left[s_{j-1}^{\;c},s_{j+1}^{\;c}\right]$ corresponding to $\left[s_{j-1}^{\;c},s_{j}^{\;p}\right)$ and $\left[s_{j}^{\;p},s_{j+1}^{\;c}\right)$. We denote the vectors of observations belonging to these two proposed segments as $y_{j}^{\;p}$ and $y_{j+1}^{\;p}$, which include $n_{j}^{\;p}$ and $n_{j+1}^{\;p}$ observations, respectively. Given $s_{\;(k)}^{\;p}$, the proposed number of frequencies $m_{j}^{\;p},m_{j+1}^{\;p}$ are set equal to the current ones $m_{j}^{\;c},m_{j+1}^{\;c}$, so that $m_{\;(k)}^{\;p}=m_{\;(k)}^{\;c}$. Similarly, the proposed pair of frequency vectors $\omega_{j}^{\;p},\omega_{j+1}^{\;p}$ is chosen equal to the current pair $\omega_{j}^{\;c},\omega_{j+1}^{\;c}$ in the corresponding segments, that is, $\omega_{\;(k)}^{\;p}=\omega_{\;(k)}^{\;c}$. The proposed vectors $\beta_{j}^{\;p},\beta_{j+1}^{\;p}$ are sampled from normal approximations to their posterior conditional distributions $\pi \;(\;\beta_{j}^{\;p}\;|\;\omega_{j}^{\;p},\;\sigma_{\;j}^{\;2c},\;m_{j}^{\;p},\;y_{j}^{\;p}\;)$ and $\pi \;(\;\beta_{j+1}^{\;p}\;|\;\omega_{j+1}^{\;p},\;\sigma_{\;j+1}^{\;2c},\;m_{j+1}^{\;p},\;y_{j+1}^{\;p}\;)$ and are accepted in a M-H step with probability

$$\begin{array}{c} \alpha =\min\{\;1,\frac{L(\;k,\;m_{\;(k)}^{\;p},\;s_{\;(k)}^{\;p},\;\theta_{\;(k)}^{\;p}\;|\;y\;)}{L(\;k,\;m_{\;(k)}^{\;c},\;s_{\;(k)}^{\;c},\;\theta_{\;(k)}^{\;c}\;|\;y\;)}\\ \;\times \frac{\pi (\;s_{\;(k)}^{\;p}\;|\;k)\;\pi (\;\theta_{\;(k)}^{\;p}\;|\;m_{\;(k)}^{\;p},\;k)}{\pi (\;s_{\;(k)}^{\;c}\;|\;k)\;\pi (\;\theta_{\;(k)}^{\;c}\;|\;m_{\;(k)}^{\;c},\;k\;)}\times \frac{\prod_{h=j}^{j+1}q\;(\;\;\beta_{h}^{\;c}\;)\;}{\prod_{h=j}^{j+1}q\;(\;\;\beta_{h}^{\;p}\;)\;}\;\}, \end{array}$$

where the likelihood is specified in Equation (4) and $q\;(\;\beta_{h}^{\;c}\;)$ and $q\;(\;\beta_{h}^{\;p}\;)$ are multivariate Gaussian as in Equation (A.3). Note that the likelihood ratio and the prior ratio differ from one only for the two segments affected by the move of the change-points. Finally, the residual variances $\sigma_{j}^{\;2p}$, $\sigma_{j+1}^{\;2p}$ are drawn from their posterior conditional distributions $\pi \;(\sigma_{j}^{\;2p}\;|\;\omega_{j}^{\;p},\;\beta_{j}^{\;p},\;m_{j}^{\;p},\;y_{j}^{\;p}),\pi \;(\sigma_{j+1}^{\;2p}\;|\;\omega_{j+1}^{\;p},\;\beta_{j+1}^{\;p},\;m_{j+1}^{\;p},\;y_{j+1}^{\;p})$ in a Gibbs step.

#### A.2.2 Between-Model Moves

Let $\xi_{\;(k^{\;\;c})}^{\;c}={\left(\;s_{\;(k^{\;\;c})}^{\;c\;\prime },\;m_{\;(k^{\;\;c})}^{\;c\;\prime },\;\theta_{\;(k^{\;\;c})}^{\;c\;\prime }\;\right)}^{\;\prime }$ and assume the Markov chain is at $\left(\;k^{\;c},\;\xi_{\;(k^{\;\;c})}^{\;c}\;\right)$. We propose a move to $\left(\;k^{\;p},\;\xi_{\;(k^{\;\;p})}^{\;p}\;\right)$ by first drawing $k^{\;p}$, followed by sampling the change-point locations $s_{\;(k^{\;\;p})}^{\;p}$. The latter involves either merging two segments (death) or splitting a segment (birth). The number of frequencies and their values in the proposed segments are selected from the current state. We draw $\beta_{\;(k^{\;\;p})}^{\;p}$ and jointly update the entire state $\left(\;k^{\;p},\;\xi_{\;(k^{\;\;p})}^{\;p}\;\right)$. Hence, we propose a move to $\left(\;k^{\;p},\;\xi_{\;(k^{\;\;p})}^{\;p}\;\right)$ by drawing from a proposal density of the form

$$\begin{array}{c} q\;(\;k^{\;p},\;\xi_{\;(k^{\;\;p})}^{\;p}\;|\;k^{\;c},\;\xi_{\;(k^{\;\;c})}^{\;c}\;)\\ \;=q\;(\;k^{\;p}\;|\;k^{\;c}\;)\times q\;(\;\xi_{\;(k^{\;\;p})}^{\;p}\;|\;k^{\;p},\;k^{\;c},\;\xi_{\;(k^{\;\;c})}^{\;c}\;)\\ \;=q\;(\;k^{\;p}\;|\;k^{\;c}\;)\;\times q\;(\;s_{\;(k^{\;\;p})}^{\;p}\;|\;k^{\;p},\;k^{\;c},\;\xi_{\;(k^{\;\;c})}^{\;c}\;)\\ \;\times q\;(\;m_{\;(k^{\;\;p})}^{\;p},\;\omega_{\;(k^{\;\;p})}^{\;p}\;|\;s_{\;(k^{\;\;p})}^{\;p},\;k^{\;p},\;k^{\;c},\;\xi_{\;(k^{\;\;c})}^{\;c}\;)\\ \;\times q\;(\sigma_{\;(k^{\;\;p})}^{\;2p}\;|\;m_{\;(k^{\;\;p})}^{\;p},\;\omega_{\;(k^{\;\;p})}^{\;p},\;s_{\;(k^{\;\;p})}^{\;p},\;k^{\;p},\;k^{\;c},\;\xi_{\;(k^{\;\;c})}^{\;c}\;)\\ \;\times q\;(\;\beta_{\;(k^{\;\;p})}^{\;p}\;|\;\sigma_{\;(k^{\;\;p})}^{\;2p},\;m_{\;(k^{\;\;p})}^{\;p},\;\omega_{\;(k^{\;\;p})}^{\;p},\;s_{\;(k^{\;\;p})}^{\;p},\;k^{\;p},\;k^{\;c},\;\xi_{\;(k^{\;\;c})}^{\;c}\;). \end{array}$$

**Birth move:** If a birth move is proposed, we have that $k^{\;p}=k^{\;c}+1$. We draw a new change-point uniformly on $f\;(s_{\;(k^{\;\;c})}^{\;c},\;\psi_{s})$, the support of $s_{\;(k^{\;\;c})}^{\;c}$ given the constraints imposed by *ψ_s_*, that is, $f\;(s_{\;(k^{\;\;c})}^{\;c},\;\psi_{s})=[1+\psi_{s},\;s_{1}^{\;c}-\psi_{s}]\;\cup \;[s_{1}^{\;c}+\psi_{s},\;s_{2}^{\;c}-\psi_{s}]\cup \;\cdots \;\cup \;[s_{k^{\;\;c}}^{\;c}+\psi_{s},\;n-\psi_{s}].$ Hence, the new proposed location ${\tilde{s}}_{j}$ is sampled from a $\text{Uniform}\;\{\;f\;(s_{\;(k^{\;\;c})},\;\psi_{s})\}$, where the proposal density is given by

(A.6) $$q\;(s_{\;(k^{\;\;p})}^{\;p}\;|\;k^{\;p},\;k^{\;c},\;\xi_{\;(k^{\;\;c})}^{\;c})=\frac{1}{\left(n-2\psi_{s}\;(k^{\;c}+1)-1\right)}.$$

As the proposed location ${\tilde{s}}_{j}$ will lie within an existing interval $\left(s_{j}^{\;c},\;s_{j+1}^{\;c}\right)$ with probability one, we can define the proposed change-points location vector as

$$s_{\;(k^{\;\;p})}^{\;p}=(s_{1}^{\;c},\ldots ,s_{j}^{\;c},{\tilde{s}}_{j},s_{j+1}^{\;c},\ldots ,s_{k^{\;\;c}}^{\;c})\prime .$$

The number of frequencies $m_{j}^{\;p},m_{j+1}^{\;p}$ corresponding to the two newly proposed segments $\left[s_{j}^{\;c},{\tilde{s}}_{j}\right)$ and $\left[{\tilde{s}}_{j},s_{j+1}^{\;c}\right)$ are set equal to the current number of frequencies on the whole segment $\left(s_{j}^{\;c},\;s_{j+1}^{\;c}\right)$. Therefore, we can construct the proposed vector of the number of frequencies $m_{\;(k^{\;\;p})}^{\;p}$ and the proposed vector of frequencies $\omega_{\;(k^{\;\;p})}^{\;p}$ as

$$\begin{array}{c} m_{\;(k^{\;\;p})}^{\;p}=(\;m_{1}^{\;c},\ldots ,m_{j-1}^{\;c},m_{j}^{\;c},m_{j}^{\;c},m_{j+1}^{\;c},\ldots ,m_{k^{\;\;c}+1}^{\;c})\prime ,\\ \omega_{\;(k^{\;\;p})}^{\;p}=(\omega_{1}^{\;c\;\prime },\ldots ,\omega_{j-1}^{\;c\;\prime },\omega_{j}^{\;c\;\prime },\omega_{j}^{\;c\;\prime },\omega_{j+1}^{\;c\;\prime },\ldots ,\omega_{k^{\;\;c}+1}^{\;c\;\prime })\prime . \end{array}$$

The proposed vector of residual variances $\sigma_{\;(k^{\;\;p})}^{\;2p}$ is

$$\sigma_{\;(k^{\;\;p})}^{\;2p}={\left(\;\sigma_{1}^{\;2c},\ldots ,\sigma_{j-1}^{\;2c},\sigma_{j}^{\;2p},\sigma_{j+1}^{\;2p},\sigma_{j+1}^{\;2c},\ldots ,\sigma_{k^{\;\;c}+1}^{\;2c}\;\right)}^{\;\prime },$$

where the residual variances $\sigma_{j}^{\;2p},\sigma_{j+1}^{\;2p}$ for the split partition are constructed following Green (1995), namely as a perturbation of the current variance $\sigma_{j}^{\;2c}$. Specifically, we draw u∼Uniform (0,1) and let $\sigma_{j}^{\;2p},\sigma_{j+1}^{\;2p}$ be deterministic transformations of $\sigma_{j}^{\;2c}$, that is

(A.7) $$\sigma_{j}^{\;2p}=\frac{u}{1-u}\sigma_{j}^{\;2c},\;\sigma_{j+1}^{\;2p}=\frac{1-u}{u}\sigma_{j}^{\;2c}.$$

Finally, the proposed vector of linear coefficients $\beta_{\;(k^{\;\;p})}^{\;p}$ is

$$\beta_{\;(k^{\;\;p})}^{\;p}={\left(\;\beta_{1}^{\;c\;\prime },\ldots ,\beta_{j-1}^{\;c\;\prime },\beta_{j}^{\;p\;\prime },\beta_{j+1}^{\;p\;\prime },\beta_{j+1}^{\;c\;\prime },\ldots ,\beta_{k^{\;\;c}+1}^{\;c\;\prime }\;\right)}^{\;\prime },$$

where the vectors $\beta_{j}^{\;p},\beta_{j+1}^{\;p}$ are drawn from normal approximations of their posterior conditional distribution, as in Section A.1.1. The proposed move to the state $\left(k^{\;p},\;\xi_{\;(k^{\;\;p})}^{\;p}\right)$ is accepted with probability

$$\begin{array}{c} \alpha =\min\{\;1,\frac{L(\;k^{\;p},\;\xi_{\;(k^{\;\;p})}^{\;p}\;|\;y\;)}{L(\;k^{\;c},\;\xi_{\;(k^{\;\;c})}^{\;c}\;|\;y\;)}\times \frac{\pi \;(k^{\;p})\;\pi \;(\xi_{\;(k^{\;\;p})}^{\;p}\;|\;k^{\;p})}{\pi \;(k^{\;c})\;\pi \;(\xi_{\;(k^{\;\;c})}^{\;c}\;|\;k^{\;c})}\\ \;\times \frac{d_{k^{\;\;p}}\cdot \frac{1}{k^{\;p}}\cdot \frac{1}{2}\cdot q\;(\beta_{j}^{\;c})}{b_{k^{\;\;c}}\cdot q\;(s_{\;(k^{\;\;p})}^{\;p})\cdot \prod_{h=j}^{j+1}q\;(\beta_{h}^{\;p})}\times \;J_{\sigma^{\;\;2}}\;\}, \end{array}$$

where the likelihood function is provided in Equation (4), $q\;(s_{\;(k^{\;\;p})}^{\;p})$ is the uniform density defined in Equation (A.6); $q\;(\beta_{h}^{\;c})$, $q\;(\beta_{h}^{\;p})$ are the multivariate normal proposal densities, and the Jacobian $J_{\sigma^{\;\;2}}$ is

$$J_{\sigma^{\;\;2}}=|\;\frac{\partial \;(\sigma_{j}^{\;2p},\sigma_{j+1}^{\;2p})}{\partial \;(\sigma_{j}^{\;2c},u)}|=2\;{\left(\sqrt{\sigma_{j}^{\;2p}}+\sqrt{\sigma_{j+1}^{\;2p}}\;\right)}^{\;2}.$$

The numerator of the proposal ratio is better understood by looking at the details of the death step, which are given below.

**Death move:** If a death step is proposed, then $k^{\;p}=k^{\;c}-1$. A candidate change-point $s_{j}^{\;c}$ to be removed is sampled uniformly from the vector of existing change-points; that is, we propose to remove $s_{j}^{\;c}$ with probability $\frac{1}{k^{\;c}}$. Then, the proposed vector of change-points locations $s_{\;(k)}^{\;p}$ is defined as

$$s_{\;(k)}^{\;p}={\left(s_{1}^{\;c},\ldots ,s_{j-1}^{\;c},s_{j+1}^{\;c},\ldots ,s_{k^{\;\;c}}^{\;c}\right)}^{\;\prime }.$$

The number $m_{j}^{\;p}$ and the vector of relevant frequencies $\omega_{j}^{\;p}$ of the newly merged segment $\left[s_{j-1}^{\;c},s_{j+1}^{\;c}\right)$ are selected by drawing an index at random from {j,j+1}, obtaining say $j^{\;*}$, and setting the proposed parameters equal to the current ones relative to the selected index. That is, we set $m_{j}^{\;p}=m_{j^{\;\;*}}^{\;c}$ and $\omega_{j}^{\;p}=\omega_{j^{\;\;*}}^{\;c}$. Hence, the proposed vectors of number of frequencies $m_{\;(k^{\;\;p})}^{\;p}$ and their values $\omega_{\;(k^{\;\;p})}^{\;p}$ are constructed as follows

$$\begin{array}{c} m_{\;(k^{\;\;p})}^{\;p}={\left(\;m_{1}^{\;c},\ldots ,m_{j-1}^{\;c},m_{j}^{\;p},m_{j+2}^{\;c},\ldots ,m_{k^{\;\;c}+1}^{\;c}\;\right)}^{\;\prime },\\ \omega_{\;(k^{\;\;p)}}^{\;p}={\left(\;\omega_{1}^{\;c\;\prime },\ldots ,\omega_{j-1}^{\;c\;\prime },\omega_{j}^{\;p\;\prime },\omega_{j+2}^{\;c\;\prime },\ldots ,\omega_{k^{\;\;c}+1}^{\;c\;\prime }\right)}^{\;\prime }. \end{array}$$

The residual variance $\sigma_{j}^{\;2p}$ of the newly merged segment is obtained by inverting the transformation of Equation (A.7). Specifically, we construct $\sigma_{j}^{\;2p}=\sqrt{\sigma_{j}^{\;2c}\;\sigma_{j+1}^{\;2c}}$ and set the proposed vector of residual variances $\sigma_{\;(k^{\;\;p})}^{\;2p}$ as

$$\sigma_{\;(k^{\;\;p})}^{\;2p}={\left(\;\sigma_{1}^{\;2c},\ldots ,\sigma_{j-1}^{\;2c},\sigma_{j}^{\;2p},\sigma_{j+2}^{\;2c},\ldots ,\sigma_{k^{\;\;c}+1}^{\;2c}\;\right)}^{\;\prime }.$$

The proposed vector of linear coefficients $\beta_{\;(k^{\;\;p})}^{\;p}$ is

$$\beta_{\;(k^{\;\;p})}^{\;p}={\left(\;\beta_{1}^{\;c\;\prime },\ldots ,\beta_{j-1}^{\;c\;\prime },\beta_{j}^{\;p\;\prime },\beta_{j+2}^{\;c\;\prime },\ldots ,\beta_{k^{\;\;c}+1}^{\;c\;\prime }\right)}^{\;\prime },$$

where the vector of coefficients $\beta_{j}^{\;p}$ is drawn from normal approximation to its posterior conditional distribution. The acceptance probability for the death step has the same form of the birth step, with the proper change of labelling of the variables, and the ratio terms inverted.
