# Supplementary material for: Bayesian Model Search for Nonstationary Periodic Time Series
Source: J Am Stat Assoc. 2019 Jul 9;115(531):1320–35. doi: 10.1080/01621459.2019.1623043 (PMC7984273; doi:10.1080/01621459.2019.1623043)
Supplement: Supplemental Material [file UASA_A_1623043_SM4900.zip › Supplemental_Material/Supplemental_Material.pdf]

Supplementary Material to “Bayesian Model Search for Nonstationary Periodic Time Series”

Beniamino Hadj-Amar, Bärbel Finkenstädt, Mark Fiecas, Francis Lévi and Robert Huckstepp *

May 16, 2019

Abstract

This document includes supplemental material to the article “Bayesian Model Search for Nonstationary Periodic Time Series”. Section 1 contains further details about the parameterization of the illustrative example and a sensitivity analysis of the prior means on the number of frequencies and change-points. Sections 2 and 3 present acceptance rates in the simulation and in the case studies and details about phase investigation. In Section 4 we discuss how the state-of-the-art methods, AutoPARM and AdaptSPEC, perform in the two case studies.

*Beniamino Hadj-Amar (E-mail: B.Hadj-Amar@warwick.ac.uk), Bärbel Finkenstädt Rand (E-mail: B.F.Finkenstadt@warwick.ac.uk), Department of Statistics, University of Warwick, Coventry CV4 7AL, UK. Francis Lévi (E-mail: F.Levi@warwick.ac.uk), Warwick Medical School, University of Warwick, Coventry CV4 7AL, UK. Robert Huckstepp (E-mail: R.Huckstepp@warwick.ac.uk), School of Life Sciences, University of Warwick, Coventry CV4 7AL, UK. Mark Fiecas (E-mail: mfiecas@umn.edu), University of Minnesota, School of Public Health, Division of Biostatistics, Minneapolis, MN 55455, USA.

1 Further Details of Illustrative Example

In this section, we provide more details about the illustrative example considered in Section 5.1 of the manuscript. In particular, we give the values of the parameterization of the study and we investigate the sensitivity of our methodology AutoNOM on the prior means λ_s and λ_ω.

1.1 Parameter Values

Table 1: Illustrative example. Parameter values for simulation from model (1) of the manuscript.

Frequencies		Linear coefficients		Trends and variances	
$\omega_{1,1}$	1/24	$\boldsymbol{\beta}_{1,1}$	(2.0, 3.0)	μ_1	.010
$\omega_{1,2}$	1/15	$\boldsymbol{\beta}_{1,2}$	(4.0, 5.0)	μ_2	.000
$\omega_{1,3}$	1/7	$\boldsymbol{\beta}_{1,3}$	(1.0, 2.5)	μ_3	-.005
$\omega_{2,1}$	1/12	$\boldsymbol{\beta}_{2,1}$	(4.0, 3.0)	σ_1^2	4.0^2
$\omega_{3,1}$	1/22	$\boldsymbol{\beta}_{3,1}$	(2.5, 4.0)	σ_2^2	3.5^2
$\omega_{3,2}$	1/15	$\boldsymbol{\beta}_{3,2}$	(4.0, 2.0)	σ_3^2	2.8^2

The value of the intercept was set to zero for every segment.

1.2 Sensitivity Analysis

To investigate the influence of the prior means λ_ω and λ_s we simulate 10 realizations from the same model and run our estimation algorithm for combinations of values for λ_ω and λ_s, ranging from 0.1 to 10.0. Table 2 shows the average posterior probability of choosing the correct model, i.e. $\hat{\pi}(k = 2, m_1 = 3, m_2 = 1, m_3 = 2 \,|\, \boldsymbol{y})$. Table 3 displays the average mean squared error

$$\text{MSE} = \frac{1}{n} \sum_{t=1}^{n} \left\{ \hat{f}_t - f_t \right\}^2,$$

to asses the distance between the true underlying signal f_t and the estimated signal $\hat{f}_t$. The latter is obtained by averaging across models of differing number of components, in contrast to model selection. Specifically, if we run our procedure for S iterations, then the estimated signal $\hat{f}_t$ is defined as

$$\hat{f}_t = \frac{1}{S} \sum_{s=1}^{S} \sum_{j=1}^{k^{(s)}+1} f(t, \boldsymbol{\beta}_j^{(s)}, \boldsymbol{\omega}_j^{(s)}) \mathbb{1}_{[t \in I_j^{(s)}]}, \quad t = 1, \dots, n, \tag{1}$$

where the superscript (s) denotes the sth sample of the Markov chain. Both analyses suggest that, for this example, the choice of the prior means λ_ω and λ_s has hardly noticeable impact on the results. However, our experience is that small values for these hyper-parameters are preferable as they prevent the algorithm from overfitting and seems to be more robust to model misspecification.

Table 2: Sensitivity analysis of illustrative example. Average probability of chosing the correct model from 10 replications with varying λ_ω and λ_s

	$\lambda_s = .1$	$\lambda_s = .2$	$\lambda_s = 0.5$	$\lambda_s = 1.0$	$\lambda_s = 2.0$	$\lambda_s = 5.0$	$\lambda_s = 10.0$
$\lambda_\omega = .1$	.98	.97	.97	.99	.93	.91	1.0
$\lambda_\omega = .2$	.99	.99	.99	.99	.99	.99	.98
$\lambda_\omega = .5$	.99	1.0	.98	.95	.98	1.0	.99
$\lambda_\omega = 1.0$	.99	.94	.93	.99	.99	.99	.99
$\lambda_\omega = 2.0$	.99	.99	.99	.99	.99	.99	.99
$\lambda_\omega = 5.0$	.97	.96	.96	.97	.97	.93	.98
$\lambda_\omega = 10.0$	.94	.95	.95	.91	.93	.95	.85

Table 3: Sensitivity analysis of illustrative example. Average MSE from 10 replications with varying λ_ω and λ_s

	$\lambda_s = .1$	$\lambda_s = .2$	$\lambda_s = .5$	$\lambda_s = 1.0$	$\lambda_s = 2.0$	$\lambda_s = 5.0$	$\lambda_s = 10.0$
$\lambda_\omega = .1$	.349	.435	.370	.416	.446	.470	.319
$\lambda_\omega = .2$	.354	.360	.378	.361	.370	.404	.394
$\lambda_\omega = .5$	.400	.347	.378	.447	.430	.346	.364
$\lambda_\omega = 1.0$	.302	.392	.400	.382	.321	.400	.337
$\lambda_\omega = 2.0$	.307	.369	.404	.340	.407	.329	.391
$\lambda_\omega = 5.0$	.360	.387	.362	.396	.346	.350	.324
$\lambda_\omega = 10.0$	.355	.386	.340	.387	.420	.393	.428

2 Acceptance Rates

Here we report the acceptance rates in the illustrative example and in the case studies. The overall acceptance rate for the simulation study is 28.3% and for the analysis of the skin temperature is 15.2%. The overall acceptance rates for the three time series of airflow traces of a rat are 24.0%, 33.8% and 33.9%, respectively. These rates are the proportion of samples accepted in all Metropolis-Hastings steps evaluated in the sampling scheme. We also report the acceptance rates for segment model and change-point model moves, grouped by within-model, birth and death steps for both simulation and case studies (see Table 4, 5, 6, 7 and 8).

Table 4: Illustrative example. Acceptance rates for segment model and change-point model moves, grouped by within-model, birth and death steps. The overall acceptance rate is 28.3%.

	Segment	Change-Point
Within	.367	.144
Birth	.003	.001
Death	.002	.001

Table 5: Analysis of skin temperature of a healthy subject. Acceptance rates for segment model and change-point model moves, grouped by within-model, birth and death steps. The overall acceptance rate is 15.2%.

	Segment	Change-Point
Within	.202	.100
Birth	.004	.004
Death	.002	.001

Table 6: Characterizing instances of sleep apnea in rodents: time series (a). Acceptance rates for segment model and change-point model moves, grouped by within-model, birth and death steps. The overall acceptance rate is 24.0. %

	Segment	Change-Point
Within	.280	.143
Birth	.002	.001
Death	.003	.001

Table 7: Characterizing instances of sleep apnea in rodents: time series (b). Acceptance rates for segment model and change-point model moves, grouped by within-model, birth and death steps. The overall acceptance rate is 33.8%.

	Segment	Change-Point
Within	.415	.151
Birth	.001	.002
Death	.001	.001

Table 8: Characterizing instances of sleep apnea in rodents: time series (c). Acceptance rates for segment model and change-point model moves, grouped by within-model, birth and death steps. The overall acceptance rate is 33.9%.

	Segment	Change-Point
Within	.282	.146
Birth	.001	.001
Death	.001	.001

3 Phase Shift

Our proposed methodology can be used to investigate phase since the sinusoidal function $f(t, \boldsymbol{\beta}_j, \boldsymbol{\omega}_j)$ that characterizes the jth segment (see Equation (2) of the manuscript) can be re-written using trigonometric identities[1] as

$$f(t, \boldsymbol{B}_j, \boldsymbol{\omega}_j, \boldsymbol{\tau}_j) = \alpha_j + \mu_j t + \sum_{l=1}^{m_j} \left(B_{j,l} \cos(2\pi\omega_{j,l} t + \tau_{j,l}) \right),$$

where $\boldsymbol{B}_j = (B_{j,1}, \ldots, B_{j,m_j})$ and $\boldsymbol{\tau}_j = (\tau_{j,1}, \ldots, \tau_{j,m_j})$. With this notation, $B_{j,l}$ is the amplitude of the frequency $\omega_{j,l}$ and $\tau_{j,l}$ is the phase shift of the corresponding frequency. The phase $\tau_{j,l}$ of a frequency of interest $\omega_{j,l}$ can be estimated in terms of the coefficients $\beta_{j,l}^{(1)}$ and $\beta_{j,l}^{(2)}$ using the following equality

$$\tau_{j,l} = \arctan\left(-\frac{\beta_{j,l}^{(2)}}{\beta_{j,l}^{(1)}} \right), \qquad -\pi \leq \tau_{j,l} \leq \pi,$$

where credible intervals can be easily obtained from the empirical percentiles of the posterior sample. In the framework of analyzing circadian biomarker data, such as body temperature, a change in acrophase may be of interest to the clinician as this may be indicative of a disruption of the bodyclock.

[1] $\cos(a \pm b) = \cos(a)\cos(b) \mp \sin(a)\sin(b)$

4 Case Studies: Comparison with Existing Methods

In this section we investigate how AutoPARM (Davis et al. 2006) and AdaptSPEC (Rosen et al. 2012), the current state-of-the-art methods, perform in the case studies (Section 6 of the manuscript). AdaptSPEC was fitted with $J = 12$ basis functions and the results shown below are conditioned on the modal number of segments, whereas AutoPARM is performed with default tuning parameters.

4.1 Analysis of Human Skin Temperature

The estimated logarithm of the time-varying spectrum of the skin temperature time series is displayed in Figure 1, for both AutoPARM (top panel) and AdaptSPEC (bottom panel). The elements of the time-varying spectrum are functions of frequency and time, and the locations of the change-points are identified visually by inspecting the abrupt changes in power over the time axis. Broadly speaking, both AutoPARM and AdaptSPEC identify five segments and show some general agreement with each other in estimating change-points and local spectra. Both methodologies, which are based on continuous spectrum models, seem to smooth the local spectra at low frequencies in a considerable way. The only frequency peak is estimated by AutoPARM in the third segment and corresponds to a cycle of approximately 1.2 hour, which finds analogies with the spectral properties of Segment 4 estimated by our proposed approach. In particular, AutoPARM identifies the spectrum of an AR(2) process with autoregressive parameters (1.55, -0.69) in that segment. However, and most importantly, both existing methods clearly fail to detect either circadian or ultradian rhythmicity which were elicited by our method (see Figure 6 and Figure 7 of the manuscript) and are to be expected as body temperature is known to be a circadian biomarker (Krauchi & Wirz-Justice 1994).

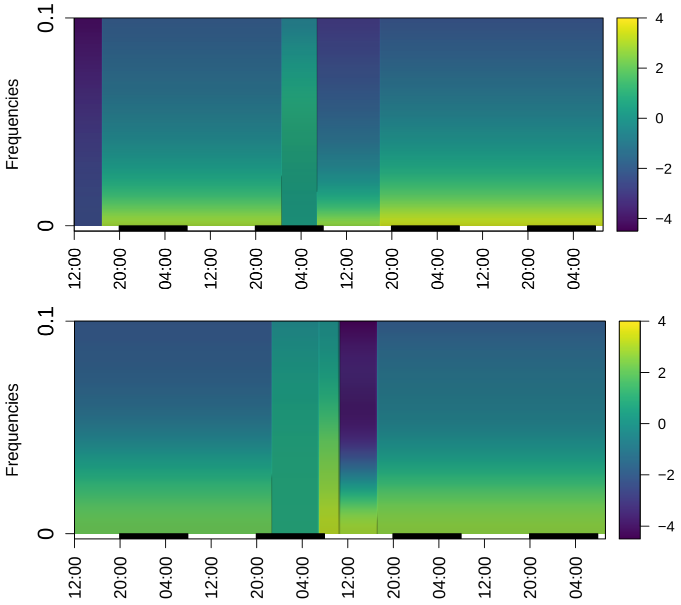

Figure 1: Estimated time-varying log spectrum for the skin temperature time series. (Top) AutoPARM. (Bottom) AdaptSPEC. Rectangles on the time axis of each plot correspond to periods from 20.00 to 8.00.

4.2 Characterizing Instances of Sleep Apnea in Rodents

The estimated time-varying spectral properties for three plethysmographic respiratory traces of the rat are displayed in Figure 2, for both AutoPARM (center panels) and AdaptSPEC (right panels). AutoPARM appears to identify fairly well changes in actions of this rat, such as (a) the alternation between sniffing and normal breathing, (b) the change from normal breathing to a spontaneous apnea, and (c) different actions of apnea, sigh and post-sigh apnea. We note that these actions were classified by eye by an experienced experimental researcher. AdaptSPEC detects changes in a satisfactory way for (a) and (c) but does not detect which distinct frequencies drive the oscillations in these data in particular as all peaks corresponding to low frequencies are

smoothed. Notice that the periodogram ordinates for these time series were approximately zero for all frequencies larger than 0.01. In addition, AdaptSPEC is not able to detect any changes from normal breathing to a spontaneous apnea since it identifies only one segment in (b). It seems that the AR building block of AutoPARM can better model peaked structures compared to the smoothing spline nature of AdaptSPEC. Generally, our method find a larger number of change-points which are associated with changes in the spectrum, as seen in Figure 8 of the main paper (right panels). For example, in (c) AutoNOM identifies different frequencies that explain the variation between Segment 3 and Segment 4, leading to the hypothesis that there might be a time changing spectrum during the occurrence of an apnea instance.

References

Davis, R. A., Lee, T. C. M. & Rodriguez-Yam, G. A. (2006), 'Structural break estimation for non-stationary time series models', *Journal of the American Statistical Association* **101**(473), 223–239.

Krauchi, K. & Wirz-Justice, A. (1994), 'Circadian rhythm of heat production, heart rate, and skin and core temperature under unmasking conditions in men', *American Journal of Physiology-Regulatory, Integrative and Comparative Physiology* **267**(3), R819–R829.

Rosen, O., Wood, S. & Stoffer, D. S. (2012), 'AdaptSPEC: Adaptive spectral estimation for nonstationary time series', *Journal of the American Statistical Association* **107**(500), 1575–1589.

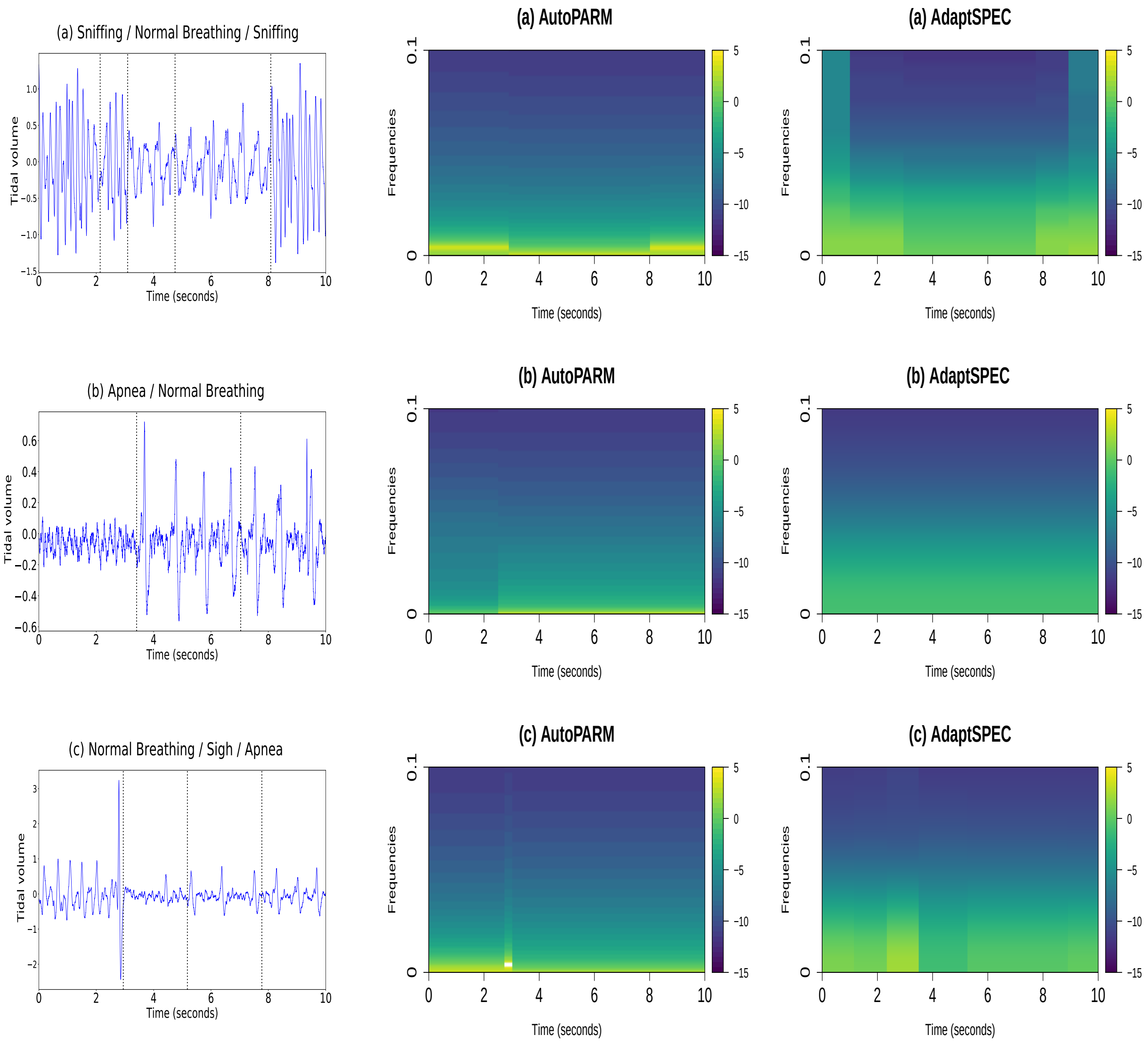

Figure 2: (Left) Plots of respiratory traces of a rat along with estimated change-points locations by AutoNOM. (a) is characterised by an alternation of sniffing and normal breathing. (b) is a plot of the trace of a spontaneous apnea, followed by normal breathing. (c) shows normal breathing followed by a sigh, and a post-sigh apnea. (Center) AutoPARM estimated time-varying log spectra for three different respiratory traces of a rat. (Right) AdaptSPEC estimated time-varying log spectra for three different respiratory traces of a rat.
